# TattleTail: A Rule-Based Tool for Identifying Canonical Pyocin-Encoding Gene Clusters in *Pseudomonas aeruginosa*

**DOI:** 10.34133/csbj.0230

**Published:** 2026-09-30

**Authors:** Rayhaan Gerard Pais, Weilian Chen, Sebastian Leptihn, Xiaoting Hua, Belinda Loh

**Affiliations:** ^1^ Department of Infection Research and Diagnostics, Fraunhofer Institute for Cell Therapy & Immunology, Leipzig, Saxony 04103, Germany.; ^2^ School of Life Sciences, Zhejiang Chinese Medical University, Hangzhou, Zhejiang 310053, China.; ^3^ Department of Infectious Diseases, Sir Run Run Shaw Hospital, Hangzhou, Zhejiang 310053, China.; ^4^ Hangzhou Digital-Micro Biotech Co., Ltd., Hangzhou, Zhejiang 311200, China.; ^5^ Department of Biochemistry and Molecular Biology, University of Southern Denmark, Odense, Funen 5230, Denmark.; ^6^ Zhejiang Provincial Engineering Research Center of Innovative Instruments for Precise Pathogen Detection, Hangzhou, Zhejiang 310016, China.

## Abstract

Tailocins are phage tail-like bacteriocins derived from prophages that have lost the ability to package viral genomes while retaining bactericidal activity against related bacterial strains. In *Pseudomonas aeruginosa*, these elements are referred to as pyocins and contribute to ecological fitness and antimicrobial applications. Despite lacking essential phage components, pyocin-encoding gene clusters share substantial sequence homology with phage tail genes, creating a major challenge for genome annotation. Current prophage prediction tools, which rely on phage gene homology, can frequently misclassify pyocins as prophages, potentially confounding genomic analyses and downstream experimental design. Here, we present TattleTail, a rule-based bioinformatic pipeline for identifying canonical pyocin-encoding gene clusters in *P. aeruginosa*. The current implementation targets the characteristic *trpE*–*trpG*-associated genomic architecture of these loci and integrates the detection of conserved pyocin-associated genes with the exclusion of canonical phage features, enabling discrimination between pyocin-encoding gene clusters and prophage regions. Evaluation across manually curated and independently selected *P. aeruginosa* genomes, together with *P. aeruginosa* genomes computationally depleted of their pyocin loci and non-*P. aeruginosa* controls, demonstrated accurate identification of canonical pyocin loci without false-positive predictions. Application to clinical isolates identified representative pyocin clusters, which were experimentally validated by mitomycin C induction, transmission electron microscopy, and bactericidal activity assays, supporting that predicted loci in these isolates correspond to functional pyocin particles. TattleTail complements existing prophage prediction tools and supports accurate annotation of phage-derived elements.

## Graphical Abstract

**Figure:**
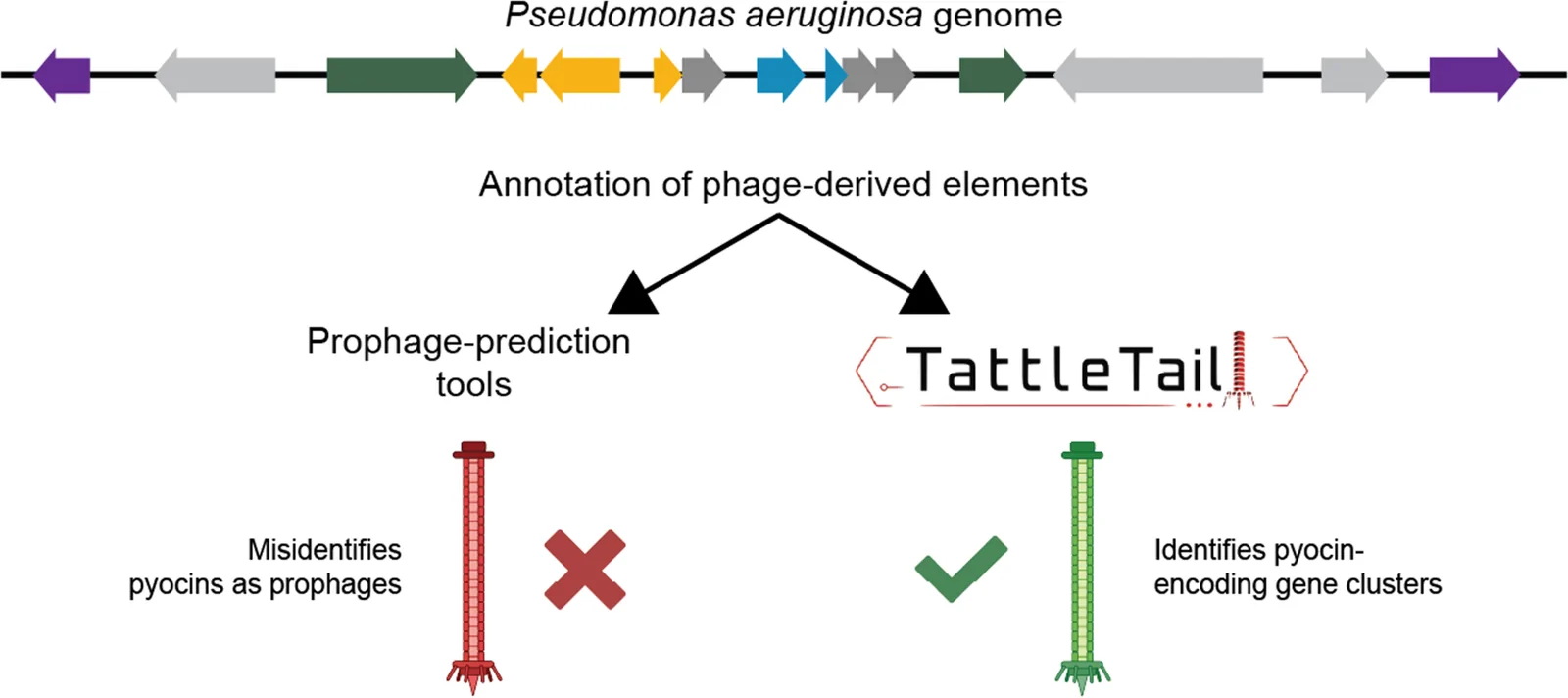

## Introduction

Bacteria employ a wide range of antagonistic strategies to compete within densely populated microbial communities. Among these, prophages and bacteriocins play key roles in shaping microbial dynamics [1,2]. Integrated prophages can confer fitness advantages to their bacterial hosts by encoding antibiotic resistance genes, toxins, and virulence genes and by providing superinfection immunity, thereby influencing host metabolism and physiology [3,4]. Upon prophage induction, bacteriophage-mediated lysis of competitor bacterial species can occur, eliminating rival strains and contributing to the shaping of the microbial community structure [5].

Bacteriocins are antimicrobial peptides, proteins, or protein complexes produced by bacteria to inhibit competing bacterial strains from occupying the same ecological niche [6]. Tailocins constitute a distinct class of bacteriocins derived from bacteriophage tails and are considered evolutionary remnants of prophages that have lost the ability to package and transmit viral genomes while retaining the capacity to kill target bacteria upon induction [7–9]. As a result, tailocins lack canonical prophage components, such as capsids, terminases, and integrases, but retain the structural and functional elements of phage tails, including tail fiber and assembly proteins [8,10]. This combination of functional divergence and genetic similarity to bacteriophages places tailocins at the interface between bacteriocins and prophages.

Tailocins produced by *Pseudomonas aeruginosa*, termed pyocins, are among the best-characterized systems of this class. They are broadly classified into R-type and F-type pyocins, which resemble contractile myovirus-like and noncontractile siphovirus-like phage tails, respectively [11], as well as soluble S-type pyocins [9]. Many *P. aeruginosa* strains harbor conserved pyocin gene clusters that encode one or more of these pyocins and are typically organized within defined genomic loci [12].

A range of bioinformatic tools have been developed for the identification and annotation of prophages in bacterial genomes, including PHASTEST [13], Phigaro [14], PhiSpy [15], VirSorter [16], Prophage Hunter [17], and Prophinder [18]. These tools have enabled large-scale and systematic identification of prophage regions across bacterial genomes, thereby facilitating studies of prophage diversity and distribution, and contributed to a deeper understanding of the role of prophages in bacterial evolution and ecology.

Despite their functional distinction from prophages, tailocins share substantial sequence homology with phage-derived genes due to their common evolutionary origin [19]. This overlap poses a fundamental challenge for genome annotation. Current prophage prediction tools rely heavily on the detection of phage-associated genes and sequence similarity to known phage proteins. As a result, tailocin-encoding gene clusters are frequently misannotated as intact or partial prophages. Such misannotation can inflate estimates of prophage abundance, distort interpretations of horizontal gene transfer and lysogeny, and confound experimental studies of bacterial competition and prophage induction. At the same time, the prevalence and diversity of tailocins are likely underestimated due to the absence of dedicated identification approaches.

At present, the identification of pyocin-encoding gene clusters in *P. aeruginosa* relies largely on manual inspection of genome annotations guided by characteristic genomic features such as conserved flanking regions, regulatory genes, and the absence of capsid-associated features. While effective, this approach is time-consuming, not scalable, and difficult to apply consistently across large genomic datasets. The absence of a dedicated, automated approach therefore prompted the development of a dedicated bioinformatic tool for the systematic identification and annotation of pyocin-encoding gene clusters.

To our knowledge, no dedicated bioinformatic tool is currently publicly available for academic use for the systematic identification of tailocin or pyocin gene clusters. Tailocin systems occur across diverse gram-negative bacteria and may possess genomic architectures distinct from the *trpE*–*trpG*-associated organization observed in *P. aeruginosa*. The present version of TattleTail was deliberately restricted to this canonical *P. aeruginosa* context, and expansion to other taxa will require taxon-specific markers and independently validated reference datasets. Here, we present TattleTail, a rule-based computational pipeline developed to automate the identification and annotation of canonical pyocin-encoding gene clusters in *P. aeruginosa*. TattleTail leverages conserved genomic markers associated with canonical pyocin loci and excludes candidate regions containing capsid-, terminase-, or integrase-like hits, thereby helping distinguish candidate canonical pyocin regions from phage-associated regions. By complementing existing prophage prediction tools, TattleTail facilitates more accurate annotation of phage-derived elements and enables large-scale analysis of pyocin distribution and diversity.

Beyond their role in bacterial competition, pyocins have attracted increasing interest as highly specific antibacterial agents [8]. Their proteinaceous and nonreplicative nature offers potential advantages over bacteriophages in therapeutic development, including improved safety and regulatory tractability. Systematic identification of pyocin systems is therefore important not only for advancing our understanding of microbial ecology and genomic evolution but also for enabling the discovery and development of targeted antibacterial strategies.

## Materials and Methods

### Architecture of TattleTail

Pyocin-encoding gene clusters in *P. aeruginosa* can be identified on the basis of certain evidence markers. First, the presence of the anthranilate synthase gene pair *trpE* and *trpG* flanking a genomic region is indicative of a known pyocin insertion hotspot [20]. Second, the candidate core must lack capsid, terminase, and integrase genes or corresponding sequence hits that are used as exclusionary features for prophage-associated regions [8]. A single hit to any of these phage-associated genes within the evaluated core is sufficient to exclude the region; no minimum number or proportion of phage-associated genes is required. Phage tail structural and assembly genes are not considered exclusionary features, as these are retained in pyocin-encoding gene clusters. Third, the candidate region must contain the pyocin-encoding regulatory genes *prtN* (transcriptional activator) and *prtR* (repressor) [21]. Fourth, the candidate region must contain both holin and endolysin genes [22].

Predicted proteins are compared with the TattleTail reference database using BLASTP. BLASTP hits meeting the specified E-value, percentage identity, alignment length, and bit-score thresholds are retained, sorted by genomic position and grouped on a per-contig basis. Retained hits located within the user-defined window are grouped into the same preliminary candidate region, while hits located on different contigs are never joined. The resulting candidate regions are then evaluated against the 4 predefined TattleTail criteria. Failure of one criterion does not prevent evaluation of the remaining criteria. A genomic region is reported as a candidate canonical pyocin-encoding gene cluster only if all 4 criteria are satisfied. It is capable of reporting more than one pyocin-encoding gene cluster per genome if multiple regions satisfy all 4 criteria. When both *trpE* and *trpG* are identified, they define the boundaries of the candidate region; the boundary genes themselves are excluded and the final reported coordinates correspond to the trimmed interflank core region. The --min-cluster-span parameter defines the minimum length required for a trimmed core region to be considered a pyocin-encoding gene cluster. The tool and usage instructions are available at https://github.com/xthua/TattleTail.

### Bacterial genomes for validating TattleTail

Genomes used to evaluate TattleTail were obtained from the National Center for Biotechnology Information RefSeq and GenBank databases. A total of 98 *P. aeruginosa* genomes (Table S1) were manually screened for the presence of pyocin-encoding gene clusters and subsequently analyzed using TattleTail. Pyocin-positive genomes were defined by manual curation based on the 4 established genomic features described above. This dataset was therefore used to evaluate the implementation consistency and recovery of the intended canonical architecture, rather than to provide a fully independent estimate of predictive sensitivity. Of these 98 *P. aeruginosa* genomes, 76 contained prophages of varying degrees of completeness classified as intact, questionable, or incomplete by PHASTEST (Table S1 and Supplementary Folder 1) [13]. Prophages of 44 of these genomes contained genes also encountered within pyocin-encoding gene clusters, including tail-associated genes, holins, and endolysins (Table S1 and Supplementary Folder 1). For the curation of these 44 genomes, tail-associated, holin, and endolysin genes were considered present when the corresponding PHASTEST annotation had an E-value <10^−50^; genes annotated as “putative” were excluded from this assessment (Table S2).

An additional 100 *P. aeruginosa* genomes (Table S3) were independently and randomly selected by a researcher at the University of Leipzig who is not involved in the development of TattleTail or the present study without prior determination of their pyocin-positive or pyocin-negative status and were subsequently analyzed using TattleTail. The resulting predictions were then manually validated based on the genomic context and the 4 established genomic features described above.

Sixty non-*P. aeruginosa Pseudomonas* genomes comprising *P. putida*, *P. protegens*, *P. chlororaphis*, and *P. fluorescens* were manually screened for the presence of containing tailocin-encoding gene clusters located between *mutS* and *cinA*, the genomic region that is the tailocin integration hotspot for these species [23,24] (Table S4), and subsequently analyzed using TattleTail.

The pyocin-encoding genomic regions between *trpE* and *trpG* were computationally excised from 2 reference genomes, *P. aeruginosa* ATCC 27853 and PAO1. The resulting genomes, designated ATCC 27853_pyocin-excised and PAO1_pyocin-excised (Supplementary Folder 1), respectively, were subsequently analyzed using TattleTail as computational negative controls.

Finally, 19 non-*Pseudomonas* genomes (Table S5) were randomly selected in GenBank and analyzed using TattleTail as additional negative controls. This dataset comprised gram-negative and gram-positive bacterial genomes as well as eukaryotic genomes.

For the validation of the tool, all genomes mentioned in this section were analyzed using the default parameters of TattleTail defined in the following section.

### Default parameters and thresholds in TattleTail

The default parameters for TattleTail are defined as follows: “E-value”, 1e−10; “pident”, 35.0; “length”, 50; “bitscore”, 50.0; “window”, 15,000; and “min-cluster-span”, 13,400.

E-value: Since TattleTail performs genome-scale screening of thousands of predicted proteins, an E-value cutoff of 1 × 10^−10^ was selected as a stringent operational threshold to minimize weak matches arising during large-scale sequence screening. This cutoff nevertheless retained the marker hits required to recover the manually identified canonical pyocin loci in all 98 *P. aeruginosa* genomes used during pipeline development.

pident: The range of 20% to 35% protein-sequence identity has been described as a “twilight zone” in which the distinction between related and unrelated proteins becomes increasingly uncertain [25]. Accordingly, the minimum identity threshold was placed at 35%, and hits below 35% identity were excluded, and identity was not interpreted independently of length, E-value, and bit score.

length and bitscore: Since the reliability of percentage identity also depends on alignment length, identity was not evaluated independently. A minimum alignment length of 50 amino acids was selected with reference to the composition of our protein database (“databse_tailocin.fasta”, Supplementary Information 1, also available via the GitHub link for TattleTail), in which the shortest reference sequence was the 82-amino acid capsid_1 protein (KGD95038.1). This threshold, therefore, permits matches to relatively compact proteins while excluding very short alignments. A minimum bit score of 50 was additionally applied as a normalized alignment-score floor.

window: The “window” value defines the maximum permitted distance between consecutive qualifying BLASTP hits on the same contig. The 15,000-bp value for this parameter was selected empirically during pipeline development using the 98 *P. aeruginosa* genomes in which pyocin-encoding gene clusters had been manually identified and which were subsequently analyzed via TattleTail. With a 10,000-bp window, the pyocin-marker hit chains in these genomes were fragmented to varying degrees, preventing the complete loci from being grouped correctly. Increasing the window to 15,000 bp retained the complete pyocin regions in all 98 genomes. Although larger windows may connect more widely separated marker hits, they may also increase the risk of merging unrelated phage-derived regions. For example, capsid-, terminase-, or integrase-like hits may be connected to a potential region and trigger the cluster-level exclusion rule. Therefore, the value of 15,000 bp was retained as a conservative default that balances locus integrity against the risk of erroneous merging. However, this parameter can also be altered from the default value, based on the needs of the user.

min-cluster-span: A “min-cluster-span” value of 13,400 bp was selected after examining the candidate core lengths recovered from the 98 *P. aeruginosa* genomes wherein pyocin-encoding gene clusters were manually identified and subsequently analyzed via TattleTail using the 15,000-bp window. The threshold was set substantially below the smallest positive core observed in this dataset. Its purpose is to suppress the output of clearly short, fragmented, and redundant hit clusters while retaining potentially shorter but otherwise complete loci, thereby improving the clarity and readability of the output. It is not intended to represent a universal biological minimum for pyocin loci.

These thresholds are used in combination and represent adjustable operational defaults rather than universal criteria for protein homology. They are the recommended parameters for the identification of canonical pyocin-encoding gene clusters using TattleTail but can be modified according to the user’s needs.

### Bacterial strains

The reference strain *P. aeruginosa* ATCC 27853 was obtained from the German Collection of Microorganisms and Cell Cultures GmbH. The 3 randomly selected *P. aeruginosa* clinical isolates from our collection, *P. aeruginosa* 003, 005, and 012 used in this study, as well as *P. aeruginosa* 008, which was not randomly selected and was specifically used as a susceptible indicator strain for bactericidal activity assays, were obtained from the University Hospital in Leipzig.

### Whole-genome sequencing of clinical isolates

Genomic DNA from clinical isolates were extracted using the Monarch Genomic DNA Purification Kit (New England Biolabs) according to the manufacturer’s instructions. Whole-genome sequencing was performed via Illumina sequencing. Reads were adapter-trimmed using Trim Galore v0.6.10 [26], normalized and error-corrected using BBnorm from BBMap v38.98 [27] (sourceforge.net/projects/bbmap/), and assembled using SPAdes v3.15.3 [28].

The BioProject (PRJDB42114) created for this study contains BioSamples SAMD01907650, SAMD01907651, and SAMD01907652, which correspond to *P. aeruginosa* 003, *P. aeruginosa* 005, and *P. aeruginosa* 012, respectively. The raw reads (sra-study) for *P. aeruginosa* 003, 005, and 012 can be accessed via the following accession number: DRP017444.

Assemblies were uploaded to the Proksee web server [29] for genome visualization and subsequently exported as concatenated single FASTA files.

The draft genome assemblies of *P. aeruginosa* 003, 005, and 012 analyzed in this study, which are provided in Supplementary Folder 2, have been submitted to the DNA Data Bank of Japan (DDBJ) and are currently awaiting accession number assignment.

The genome sequences of *P. aeruginosa* ATCC 27853 (NZ_CP101912.1) and *P. aeruginosa* PAO1 (NC_002516.2) were obtained from RefSeq.

### Prediction of prophages in clinical genomes

Draft genome assemblies were analyzed using 3 prophage prediction tools, namely, PHASTEST (version 3.0) [13], Phigaro (version 2.4.0) [14], and VirSorter (version 2.2.4) [16]. PHASTEST analysis was performed using the deep annotation mode, while Phigaro and VirSorter analyses were performed using their respective default parameters. Predicted regions were manually evaluated using the criteria described above to determine whether they correspond to pyocin-encoding loci rather than prophages. The genomes of *P. aeruginosa* ATCC 27853 and *P. aeruginosa* PAO1, which served as reference strains in this study, were also analyzed using all 3 tools under the same conditions. Detailed outputs of the analyses via prophage tools can be found in Supplementary Folder 3.

### Prediction of pyocin-encoding genes clusters in clinical genomes using TattleTail

TattleTail was applied to the reference *P. aeruginosa* ATCC 27853 and *P. aeruginosa* PAO1 genomes and to clinical isolate genomes using the default parameters of TattleTail: “E-value”, 1e−10; “pident”, 35.0; “length”, 50; “bitscore”, 50.0; “window”, 15,000; and “min-cluster-span”, 13,400. Regions satisfying all 4 predefined criteria were classified as pyocin-encoding gene clusters.

For each genome analyzed, TattleTail generated a human-readable text report, a structured JSON report and, for each reported candidate, gene-level tables and annotated GFF3 and GenBank files containing predicted gene products and genomic coordinates.

### Manual determination of the R-pyocin type

Pyocin types were determined by pairwise alignment of tail fiber protein sequences using EMBOSS Stretcher [30]. The tail fiber protein sequences in the predicted pyocin-encoding gene clusters were compared to those from representative reference sequences for each pyocin-type: R1 (WHT11591.1) from *P. aeruginosa* S14 [12], R2 (NP_249311.1) from *P. aeruginosa* PAO1 [31], and R5 (DBA05840.1) from *P. aeruginosa* isolate 3-5 [11]. Pyocin classification was performed independently of TattleTail predictions.

### Annotation of identified pyocin-encoding gene clusters via TattleTail

Identified pyocin-encoding gene clusters were annotated as part of the TattleTail pipeline, facilitated by Bakta (version 1.12.0) [32,33]. For each candidate cluster, the entire input genome sequence was first annotated with Bakta (version 1.12.0). The core pyocin cluster region was then extracted according to the precise genomic coordinates determined by TattleTail. To improve functional annotation accuracy, a custom protein database (database_tailocin.fasta, Supplementary Information 1 and also available in the GitHub repository) containing verified pyocin-related proteins was supplied to Bakta via the --proteins parameter. This approach avoids the boundary drift commonly observed when annotating short extracted fragments and ensures more reliable gene product assignments, especially for key genes such as holin, endolysin, *prtN*, and *prtR*.

### Induction and tangential flow filtration of pyocins

Single colonies of the reference strain *P. aeruginosa* ATCC 27853 and each clinical *P. aeruginosa* isolate were grown overnight in 5 ml of lysogeny broth (LB) at 37 °C with shaking at 220 rpm. Cultures were diluted 1:20 (v/v) into 100 ml of LB and grown under the same conditions to OD_600_ ≈ 0.60. Pyocin production was induced as previously described [34]. Briefly, mitomycin C was added to a final concentration of 3 μg/ml, followed by incubation for ~18 h under the same conditions. Cultures were treated with 10 μl of chloroform and centrifuged at 8,000 × *g* for 30 min at 4 °C, and supernatants were filtered through 0.22-μm filters. Uninduced controls were prepared under identical conditions, omitting the addition of mitomycin C.

Buffer exchange was performed using tangential flow filtration (TFF; μPulse TFF system) with a 300-kDa-molecular-weight polyethersulfone filter. Samples were exchanged into storage buffer (10 mM Tris–HCl, pH 7.0; 10 mM MgSO_4_). Uninduced control samples were processed identically.

### Transmission electron microscopy

Samples were prepared as described previously [34]. Briefly, pyocin preparations were absorbed onto thin carbon films, washed, negatively stained with 2% (w/v) aqueous uranyl acetate solution (pH 5.0), and examined with an EM 910 transmission electron microscope (Carl Zeiss, Oberkochen, Germany) at an acceleration voltage of 80 kV. Size determination of particles was performed using the ITEM software (Olympus Soft Imaging Solutions, Münster).

### Soft-agar overlay assay

Indicator strain cultures (*P. aeruginosa* 008) were grown in LB at 37 °C with shaking at 220 rpm to an OD_600_ of 0.60. Soft-agar overlays were prepared by mixing cultures with 0.7% (w/v) LB soft agar at a 1:10 (v/v) ratio and pouring onto LB agar plates. After solidification, 2 μl of serially diluted TFF-processed pyocin preparations (10^0^ to 10^−4^ in storage buffer) were spotted onto the bacterial lawn and incubated at 37 °C for 16 h. TFF-processed uninduced preparations were diluted and spotted in the same manner.

For proteinase K treatment controls, proteinase K was added to TFF-processed pyocin preparations at a final concentration of 0.2 mg/ml and incubated at 37 °C for 1 h. These preparations were then serially diluted and spotted on a lawn of *P. aeruginosa* 008 as described above.

For self-susceptibility testing, bacterial lawns of *P. aeruginosa* 003, 005, and 012 were created as described above. Serial dilutions of each TFF-processed pyocin preparation were then spotted on their respective producer strain.

Plates were incubated at 37 °C for 16 h before zones of growth inhibition were recorded. All assays were performed in biological triplicate.

### Verification of the lack of amplification potential

Lack of amplification potential was verified as previously described [34]. Pyocin preparations were added to *P. aeruginosa* 008 cultures (OD_600_ of 0.60) at a 1:100 (v/v) ratio and incubated for 16 h at 37 °C with shaking at 220 rpm. Following incubation, cultures were centrifuged at 8,000 × *g* for 10 min at 4 °C. Supernatants were treated with chloroform and centrifuged under identical conditions, and clarified supernatants were collected. Samples were serially diluted (10^0^ to 10^−3^) in LB, and 2-μl aliquots of each dilution were spotted onto *P. aeruginosa* 008 lawns prepared as described in the “Soft-agar overlay assay” section.

In parallel, pyocin preparations were diluted 1:100 (v/v) in LB and immediately subjected to serial dilution (10^0^ to 10^−3^). Aliquots (2 μl) were spotted on *P. aeruginosa* 008 lawns. Plates were incubated at 37 °C for 16 h. Activity before and after the amplification assay was subsequently compared. All experiments were performed in biological triplicates.

## Results

### Prophage prediction tools misclassify pyocin gene clusters as prophages

We previously reported that the prophage prediction tool PHASTEST misannotated a pyocin biosynthetic gene cluster in a reference *P. aeruginosa* strain as an intact prophage despite the absence of capsid and genome-packaging functions [34]. Here, we investigated whether misclassification of pyocin loci also occurs with other prophage prediction tools by analyzing 3 clinical *P. aeruginosa* isolates and the reference strains *P. aeruginosa* ATCC 27853 and *P. aeruginosa* PAO1 using PHASTEST, Phigaro, and VirSorter.

Across these genomes, the pyocin loci in *P. aeruginosa* ATCC 27853, PAO1, 003, and 012 were classified as intact prophages and as a questionable prophage in *P. aeruginosa* 005 when analyzed using PHASTEST (Table S6; detailed PHASTEST output in Supplementary Folder 3). When analyzed using Phigaro, pyocin loci were classified as prophages in all 5 cases (Table S7; detailed Phigaro output in Supplementary Folder 3). Analysis using VirSorter showed that the pyocin loci in *P. aeruginosa* ATCC 27853, PAO1, 003, and 012 were classified as prophages, whereas the pyocin locus in *P. aeruginosa* 005 was not classified as a prophage at all (Table S8; detailed VirSorter output in Supplementary Folder 3).

These results demonstrate that pyocin-encoding loci can be misclassified as prophages across multiple general prophage prediction tools and motivated the development of TattleTail, a dedicated approach for identifying pyocin-encoding clusters, thus helping distinguish them from prophage regions.

### Development of the TattleTail pyocin prediction pipeline

TattleTail was implemented as a rule-based pipeline that identifies pyocin-encoding regions using conserved genomic features together with the absence of canonical phage genes. The design of the pipeline is guided by the defining characteristics of pyocin loci in *P. aeruginosa*, which are canonically located between conserved tryptophan biosynthesis genes and lack genes required for phage particle formation.

For a region to be detected as a candidate canonical pyocin-encoding gene cluster by TattleTail, all 4 previously defined criteria must be met. The genomic region between the flanking genes *trpE* and *trpG* defines the locus known to harbor pyocin gene clusters; therefore, candidate regions must be flanked by this gene pair. Candidate regions must not contain capsid-, terminase-, or integrase-like hits under the selected BLASTP thresholds, as these features are indicative of intact prophages rather than pyocin systems. A single such hit is sufficient to exclude the region. The region must also contain the regulatory genes *prtN* and *prtR*, which define the canonical regulatory architecture of pyocin loci. Finally, the region must possess the lytic module genes holin and endolysin, which are essential for pyocin release. Although spanins are commonly associated with phage-mediated cell lysis in gram-negative bacteria, they have not been reported as components of pyocin-encoding gene clusters in *P. aeruginosa*. Consistent with this, no spanin genes were identified within the pyocin-encoding gene clusters analyzed during the development and validation of TattleTail. Therefore, spanins were not incorporated as an identification criterion in the TattleTail decision framework. Failure of one criterion does not prevent evaluation of the remaining criteria, and only regions that satisfy all 4 criteria are classified as pyocin-encoding clusters and are reported as the genomic region between the *trpE* and *trpG* genes. This decision framework is summarized in Fig. 1.

**Fig. 1.**
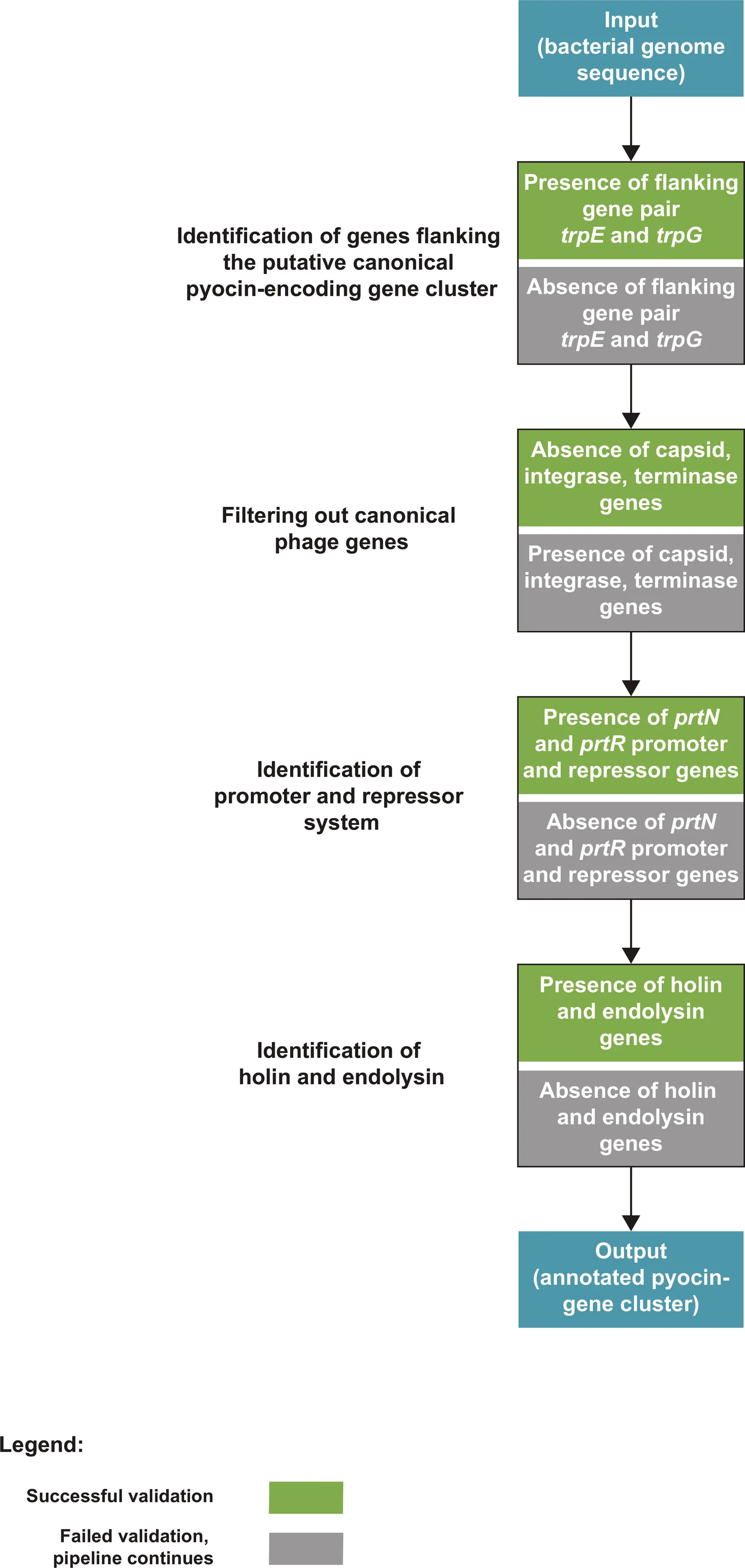
Decision framework of TattleTail: The flowchart represents the minimal combination of flanking, regulatory, and lytic markers required for a pyocin-encoding gene cluster while explicitly filtering out regions with residual intact phage structural signatures.

TattleTail generates structured reports for each genome that summarize predicted pyocin-encoding regions at both global and cluster levels. At the global level, reports indicate the presence of characteristic features, including *trpE* and *trpG* regions that flank an identified cluster, regulatory genes (*prtN* and *prtR*), and lysis genes (holin and endolysin). Cluster-level outputs detail each preliminary hit cluster, including contig identifiers, genomic coordinates of the trimmed core cluster, and stepwise evaluation outcomes for each diagnostic criterion, together with a final verdict line that classifies the candidate region.

For each predicted cluster, TattleTail generates an annotation report, which is an ordered list of genes including the gene products and genomic positions. This tabulated gene list is compatible with external genome visualization and annotation platforms such as Artemis [35], JBrowse [36], geneviewer [37], and IGV [38,39].

This decision framework enables the systematic and reproducible identification of pyocin-encoding gene clusters.

### TattleTail accurately identifies canonical pyocin-encoding gene clusters in *P. aeruginosa* genomes

TattleTail was initially evaluated using a curated benchmark dataset comprising 98 unique *P. aeruginosa* genome assemblies containing manually identified pyocin-encoding gene clusters and 19 non-*P. aeruginosa* bacterial genomes as negative controls (Tables S1 and S5, respectively). Pyocin-encoding gene clusters were identified in all 98 *P. aeruginosa* genomes, while no candidate regions were detected in any of the 19 non-*P. aeruginosa* genomes, indicating high sensitivity for pyocin loci in *P. aeruginosa* and high specificity against unrelated bacterial genomes (Tables S1 and S5; detailed TattleTail output in Supplementary Information 2A and B).

Of the 98 *P. aeruginosa* genomes, 76 contained prophages classified by PHASTEST as intact, questionable, or incomplete. Among these, 44 contained prophage regions encoding tail-associated proteins, holins, and/or endolysins, which are also characteristic components of pyocin-encoding gene clusters (Tables S1 and S2 and Supplementary Folder 1). Despite these shared features, TattleTail identified the pyocin-encoding gene clusters without misidentifying the prophage regions as pyocins (Table S1).

All predicted clusters consistently satisfied the 4 diagnostic criteria defined in the decision framework, namely, the presence of *trpE* and *trpG*, *prtN* and *prtR*, and lysis genes, together with the absence of capsid, terminase, and integrase hits. It is important to note that TattleTail always performs validation for all 4 criteria, even if the validation of one of the preceding criteria fails. This can be observed in the detailed TattleTail output of the 98 *P. aeruginosa* genomes with manually identified pyocin-encoding gene clusters, for example, in sample #13, cluster #2, and in sample #28, cluster #2, wherein the validation of the *trpE* and *trpG* genes, the *prtN* and *prtR* genes, and absence of canonical phage genes failed, but the validation of the holin and endolysin genes was successful (Supplementary Information 2A). However, for TattleTail to report a pyocin-encoding gene cluster, all 4 criteria must be validated. Inspection of TattleTail reports further showed that predicted loci were supported by high-confidence homology matches for pyocin-associated genes, with high percentage identities and low E-values observed for *prtN*, *prtR*, holin, and endolysin across isolates.

To further evaluate TattleTail using *P. aeruginosa* genomes that were not preselected based on the presence of pyocin-encoding gene clusters, an additional 100 *P. aeruginosa* genomes were independently and randomly selected and analyzed. TattleTail identified 95 pyocin-encoding gene clusters among these genomes (Table S3; detailed TattleTail output in Supplementary Information 2C). Manual validation of the resulting predictions confirmed that all 95 predicted clusters were canonical pyocin-encoding gene clusters with no false-positive predictions. From this independently curated set of 100 *P. aeruginosa* genomes, TattleTail did not predict a pyocin-encoding gene cluster in 5 genomes (Table S3, cases #10, #12, #15, #19, and #66). These 5 cases prompted further investigation which showed that regions in these genomes may represent noncanonical pyocin-like-encoding gene clusters. Based on the default parameters of TattleTail used for the analysis, the regions were not reported as pyocin-encoding gene clusters, but upon altering computational parameters, 4 of the 5 could be considered potential pyocin-encoding gene clusters. The detailed reasoning behind the results of each of the 5 cases, as well as the risks associated with altering computational rules and achieving a positive result, is discussed in the “Limitations” section of this article.

The performance of TattleTail against tailocin systems from other *Pseudomonas* species was additionally evaluated using 60 non-*P. aeruginosa Pseudomonas* genomes comprising *P. putida*, *P. protegens*, *P. chlororaphis*, and *P. fluorescens* (Table S4; detailed TattleTail output in Supplementary Information 2D). These genomes contained tailocin-encoding gene clusters located between *mutS* and *cinA*, rather than between *trpE* and *trpG*. TattleTail did not identify the tailocin-encoding gene clusters in any of the 60 genomes, consistent with the current *P. aeruginosa*-specific scope of the tool.

Finally, as computational negative controls, TattleTail was evaluated using the *P. aeruginosa* ATCC 27853 and PAO1 reference genomes in which the pyocin-encoding genomic region between *trpE* and *trpG* was computationally excised. No pyocin-encoding gene clusters were identified in either ATCC 27853_pyocin-excised or PAO1_pyocin-excised genomes, and no alternative genomic regions fulfilled all 4 criteria required for pyocin identification (Supplementary Information 2E).

Detailed TattleTail outputs and run reports for all genomes discussed in this section are available in Supplementary Information 2.

These results indicate complete recovery of pyocin loci in the positive dataset and no false-positive predictions in the negative dataset, demonstrating that the knowledge-driven TattleTail framework reliably identifies pyocin clusters in *P. aeruginosa* genomes. Furthermore, the extensive validation analyses demonstrate that TattleTail distinguishes pyocin-encoding gene clusters from prophage regions containing shared phage-derived features, while the analysis of tailocin-containing non-*P. aeruginosa Pseudomonas* genomes further defines the current taxonomic and genomic scope of the tool.

### *In silico* identification of pyocin-encoding gene clusters in clinical *P. aeruginosa* isolates using TattleTail

Having established the performance of TattleTail, we next applied the pipeline to the reference strains *P. aeruginosa* ATCC 27853 and *P. aeruginosa* PAO1 and 3 clinical isolates collected at the Leipzig University Hospital. Apart from *P. aeruginosa* PAO1 (Table S1, #1), these genomes were not included in the benchmark dataset and represent independent test cases.

In all genomes, TattleTail identified a single candidate region located between the *trpE* and *trpG* genes. Each predicted region satisfied the defined pyocin criteria including the presence of regulatory, structural, and accessory components. The resulting cluster reports for each representative isolate contained gene organizations consistent with previously described [12] R-type and F-type pyocin loci, including ordered arrangements of regulatory genes, tail-associated structural components, and accessory genes with annotated functions and genomic coordinates for each predicted region.

In contrast, PHASTEST, Phigaro, and VirSorter identified multiple putative prophage regions in the same genomes, as described in the section “Prophage prediction tools misclassify pyocin gene clusters as prophages” and consistently classified the TattleTail-predicted pyocin loci as prophages (Table 1 and Tables S6 to S8). However, it is noteworthy to point out that VirSorter did not misannotate the pyocin-encoding gene cluster as a prophage in the case of *P. aeruginosa* 005.

**Table 1.** TattleTail prediction of pyocin-encoding gene clusters in *Pseudomonas aeruginosa* genomes

Bacterial strain	Number of candidate regions	Region length	Region position
*P. aeruginosa* ATCC 27853	1	17.13 kb	679,269–696,399
*P. aeruginosa* PAO1	1	31.02 kb	672,459–703,476
*P. aeruginosa* 003	1	17.00 kb	6,283,495–6,300,498
*P. aeruginosa* 005	1	17.53 kb	3,828,968–3,846,494
*P. aeruginosa* 012	1	31.02 kb	403,320–434,337

These results show that TattleTail consistently identifies pyocin-encoding gene clusters in independent clinical *P. aeruginosa* genomes. The detailed TattleTail outputs for all 5 samples can be found in Supplementary Information 2F.

### TattleTail provides gene and protein annotation of pyocin-encoding clusters

Having demonstrated that TattleTail accurately predicts pyocin-encoding gene clusters, we applied the tool to the genomes of the reference strains *P. aeruginosa* ATCC 27853 and *P. aeruginosa* PAO1, both of which possess experimentally characterized pyocin systems [10,34], to illustrate its annotation capabilities. PAO1 was also included in the dataset used to validate TattleTail (sample #1 in Supplementary Information 2A), where a pyocin-encoding gene cluster was manually identified prior to analysis via TattleTail. The detailed annotation results of these 2 genomes are provided in Tables 2 and 3, and Supplementary Information 2G.

**Table 2.** Annotation of genes in the pyocin gene cluster of *Pseudomonas aeruginosa* ATCC 27853 carried out via TattleTail (pyocin gene cluster coordinates identified by TattleTail: 679,269 to 696,399)

#	Gene	Gene product	Genomic position
1	*prtN_1*	prtN (*P. aeruginosa*)	Complement 679,586–679,900
2	*prtR_1*	prtR (*P. aeruginosa*)	Complement 680,000–680,770
3	*FJGENJ_00636*	Hypothetical protein	Complement 680,855–681,025
4	*ptrB*	Repressor, PtrB	681,228–681,428
5	*FJGENJ_00638*	Hypothetical protein	681,476–681,835
6	*holin_6*	Holin (*P. aeruginosa*)	682,294–682,647
7	*FJGENJ_00640*	Phage protein	682,669–683,184
8	*FJGENJ_00641*	Pyocin	683,181–683,738
9	*FJGENJ_00642*	IraD/Gp25-like domain-containing protein	683,891–684,217
10	*FJGENJ_00643*	Baseplate protein J	684,214–685,101
11	*FJGENJ_00644*	Bacteriophage protein	685,094–685,627
12	*FJGENJ_00645*	Tail fiber protein	685,629–687,734
13	*FJGENJ_00646*	Phage tail protein	687,742–688,182
14	*FJGENJ_00647*	Putative prophage major tail sheath protein	688,225–689,385
15	*FJGENJ_00648*	Major tail tube protein	689,398–689,901
16	*FJGENJ_00649*	Phage tail assembly protein	689,916–690,260
17	*FJGENJ_00650*	Phage tail tape measure protein	690,430–692,667
18	*FJGENJ_00651*	Phage tail protein	692,677–693,549
19	*FJGENJ_00652*	Phage tail protein	693,524–693,730
20	*FJGENJ_00653*	Phage protein D	693,788–694,777
21	*endolysin_1*	Endolysin (*P. aeruginosa*)	694,810–695,439
22	*FJGENJ_00655*	DUF2802 domain-containing protein	695,436–695,798
23	*FJGENJ_00656*	Lipoprotein	695,795–696,052

**Table 3.** Annotation of genes in the pyocin gene cluster of *Pseudomonas aeruginosa* PAO1 carried out via TattleTail (pyocin gene cluster coordinates identified by TattleTail: 672,459 to 703,476)

#	Gene	Gene product	Genomic position
1	*prtN_1*	prtN (*P. aeruginosa*)	Complement 672,777–673,091
2	*prtR_1*	prtR (*P. aeruginosa*)	Complement 673,191–673,961
3	*NFOBNJ_00631*	Hypothetical protein	Complement 674,046–674,216
4	*ptrB*	Repressor, PtrB	674,419–674,619
5	*NFOBNJ_00633*	Hypothetical protein	674,667–675,026
6	*holin_6*	Holin (*P. aeruginosa*)	675,486–675,839
7	*NFOBNJ_00635*	Phage protein	675,861–676,376
8	*NFOBNJ_00636*	Pyocin	676,373–676,930
9	*NFOBNJ_00637*	IraD/Gp25-like domain-containing protein	677,083–677,409
10	*NFOBNJ_00638*	Baseplate protein J	677,406–678,293
11	*NFOBNJ_00639*	Bacteriophage protein	678,286–678,819
12	*NFOBNJ_00640*	Tail fiber protein	678,821–680,896
13	*NFOBNJ_00641*	Putative prophage major tail sheath protein	681,394–682,554
14	*NFOBNJ_00642*	Major tail tube protein	682,567–683,070
15	*NFOBNJ_00643*	Phage tail assembly protein	683,085–683,429
16	*NFOBNJ_00644*	Phage tail tape measure protein	683,599–685,836
17	*NFOBNJ_00645*	Phage tail protein	685,846–686,718
18	*NFOBNJ_00646*	Phage tail protein	686,693–686,899
19	*NFOBNJ_00647*	Phage protein D	686,957–687,946
20	*endolysin_1*	Endolysin (*P. aeruginosa*)	687,979–688,608
21	*NFOBNJ_00649*	DUF2802 domain-containing protein	688,605–688,967
22	*NFOBNJ_00650*	Lipoprotein	688,964–689,221
23	*NFOBNJ_00651*	Phage tail protein	689,537–690,031
24	*NFOBNJ_00652*	Phage tail assembly chaperone	690,043–690,390
25	*NFOBNJ_00653*	Hypothetical protein	690,420–690,674
26	*NFOBNJ_00654*	Bacteriophage tail tape measure C-terminal domain-containing protein	690,721–692,556
27	*NFOBNJ_00655*	Hypothetical protein	692,549–692,890
28	*NFOBNJ_00656*	Phage minor tail protein L	692,898–693,593
29	*NFOBNJ_00657*	NlpC/P60 domain-containing protein	693,596–694,366
30	*NFOBNJ_00658*	Tail assembly protein	694,421–695,023
31	*NFOBNJ_00659*	Putative phage-related protein, tail component	695,082–698,696
32	*NFOBNJ_00660*	Hypothetical protein	Complement 699,125–699,265
33	*NFOBNJ_00661*	Phage tail protein	699,744–700,835
34	*NFOBNJ_00662*	Hypothetical protein	700,835–701,170
35	*NFOBNJ_00663*	Tail fiber assembly protein	701,151–701,381
36	*NFOBNJ_00664*	Phage tail protein	701,477–702,529
37	*NFOBNJ_00665*	Lipoprotein	702,529–702,831
38	*NFOBNJ_00666*	Tail fiber assembly protein	702,828–703,058

In both genomes, TattleTail identified a single pyocin-encoding gene cluster located between the *trpE* and *trpG* genes, consistent with the conserved insertion site of the pyocin loci in *P. aeruginosa*. The predicted clusters in ATCC 27853 and PAO1 were 17.13 and 31.02 kb, respectively. This difference reflected their gene composition: the shorter ATCC 27853 region contained an R-type architecture, whereas the larger PAO1 region contained both R- and F-type pyocin gene clusters and an expanded repertoire of tail-associated and assembly-related genes. Annotation of these regions revealed the presence of the canonical regulatory genes *prtN* and *prtR*, followed by genes encoding tail-associated structural components, including baseplate, tail sheath, and tail fiber proteins, as well as accessory proteins and the lysis module genes (holin and endolysin). More generally, the approximately 17-kb and 29- to 31-kb candidate-size ranges observed across the dataset may reflect differences in structural-gene content or composite pyocin architectures; however, region length alone is insufficient to assign pyocin type or biological function.

The gene organization and function composition of the predicted clusters were consistent with known R-type and F-type pyocin systems, demonstrating that TattleTail not only identifies pyocin loci but also provides detailed and biologically coherent annotation of the encoded gene clusters in well-characterized reference strain genomes.

### Induction, morphology validation, and qualitative activity of pyocins identified in clinical *P. aeruginosa* strains

To test whether the pyocin-encoding loci identified by TattleTail in the Leipzig clinical isolates give rise to functional tailocin particles, pyocin production was induced with mitomycin C and the resulting cell-free supernatants were analyzed for particle morphology and bactericidal activity (Fig. 2A and B).

**Fig. 2.**
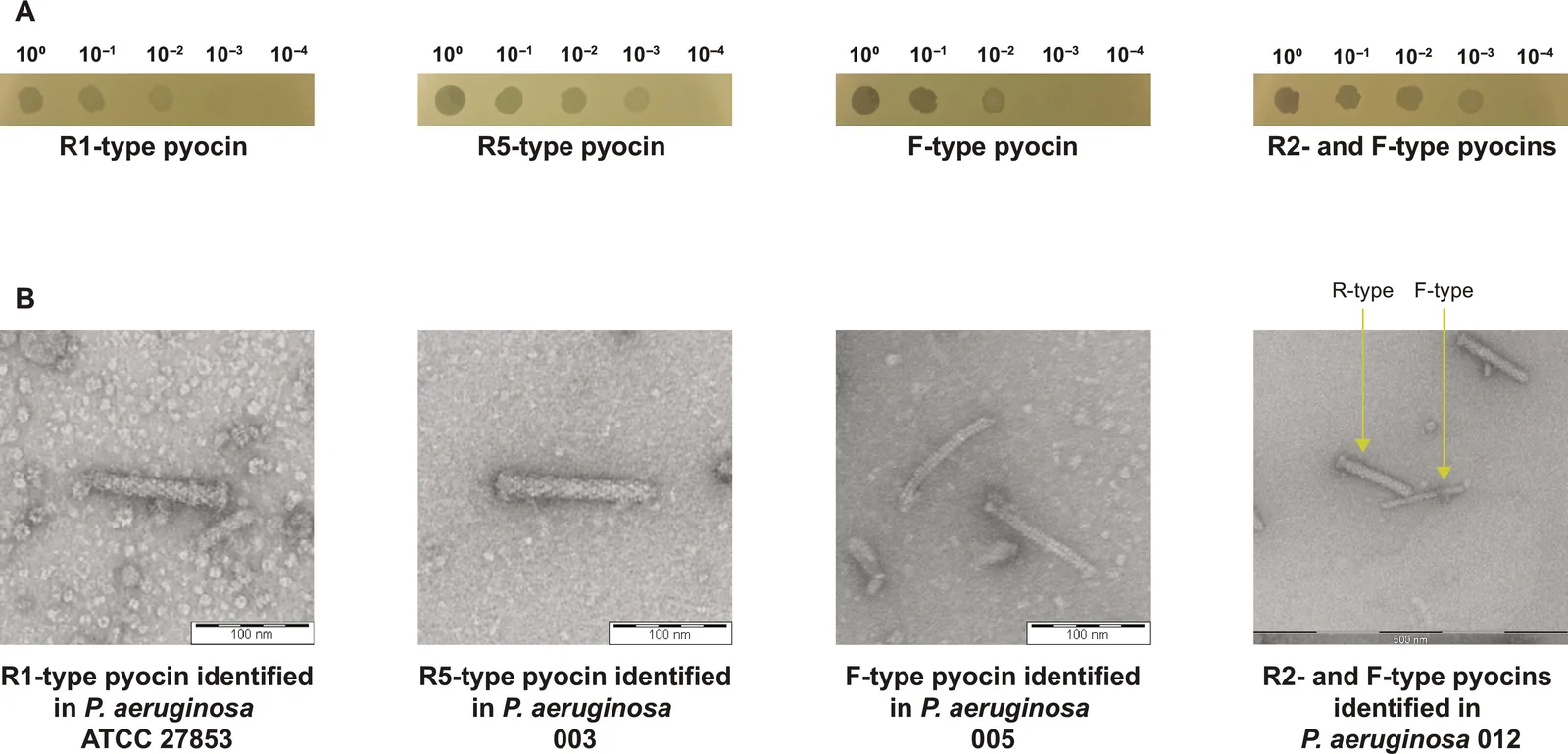
Pyocins identified in clinical *Pseudomonas aeruginosa* isolates using TattleTail. (A) Serial dilution spot test assays of representative pyocins against the target strain *P. aeruginosa* 008 (spot test for noninduced controls in Fig. S1). (B) Transmission electron micrographs showing the morphologies of identified and induced pyocins. Scale bars for each micrograph can be used as a reference for pyocin particle size.

Transmission electron microscopy of the cell-free supernatants revealed structures characteristic of phage tail-like bacteriocins, including contractile tail particles consistent with R-type pyocins and noncontractile tail particles consistent with F-type pyocins (Fig. 2B). No phage heads or DNA-containing capsids were observed, consistent with the absence of capsid and terminase genes in the corresponding genomic loci and supporting the absence of intact prophages.

Activity assays using the susceptible *P. aeruginosa* indicator strain, *P. aeruginosa* 008, showed clear zones of growth inhibition for supernatants from induced cultures (Fig. 2A), whereas noninduced controls exhibited substantially reduced activity (Fig. S1), indicating low-level basal induction in the absence of mitomycin C.

Bactericidal activity persisted following proteinase K treatment and was comparable to that of untreated samples (Fig. S2A), indicating that the observed activity was not attributable to protease-sensitive S-type pyocins.

The susceptibility of producer strains to their endogenous pyocins was evaluated. *P. aeruginosa* 003, 012, and ATCC 27853 showed no detectable self-susceptibility. In contrast, *P. aeruginosa* 005 exhibited susceptibility to its own pyocin (Fig. S2B). Self-susceptibility to endogenous R1 pyocins has been reported in *P. aeruginosa* [21], indicating that resistance to self-produced pyocins is not universal. Our observation in *P. aeruginosa* 005 complements this finding and suggests that, like for R-type pyocins, self-susceptibility may also apply to F-type pyocins.

The strains used in this study are also predicted to harbor multiple prophage regions (Tables S6 to S8). To ensure that the observed bactericidal activity was not attributable to a replicative agent, such as an induced prophage, but instead to a nonreplicative agent, i.e., pyocins, amplification potential was assessed. Supernatants collected following the amplification assay showed no increase in detectable bactericidal activity across serial dilutions compared to pyocin activity prior to the assay (Fig. S2C). This indicates that no amplification of the active agents occurred, demonstrating that the observed results can be attributed to nonreplicative bactericidal agents, consistent with pyocins.

The strains used in this study are also predicted to harbor multiple prophage regions (Tables S6 to S8). To ensure that the observed bactericidal activity was not attributable to a replicative agent, such as an induced prophage, but instead to a nonreplicative agent, i.e., pyocins, amplification potential was assessed. Supernatants collected following the amplification assay showed no increase in detectable bactericidal activity across serial dilutions compared to pyocin activity prior to the assay (Fig. S2C). This indicates that no amplification of the active agents occurred, demonstrating that the observed results can be attributed to nonreplicative bactericidal agents, consistent with pyocins.

Together, these results demonstrate that the pyocin loci present in the reference strain ATCC 27853 and the 3 clinical isolates predicted by TattleTail correspond to expressed and functional pyocins, providing experimental support for the biological relevance of the TattleTail predictions.

## Limitations

TattleTail is naturally subject to certain limitations. In its current and first iteration, the tool identifies canonical pyocin loci in *P. aeruginosa* based on their characteristic genomic location and genomic organization, with clusters located between the *trpE* and *trpG* genes. Consequently, pyocin loci occurring outside or substantially deviating from this genomic context may not be detected. An example of such a deviation, in terms of genomic organization and proximity between genes, is observed in 5 genomes, part of the 100 independently curated *P. aeruginosa* genomes (Table S3, #10, NZ_CP029090.1; #12, NZ_CP021775.1; #15, NZ_CP194326.1; #19, NZ_CP081202.1; and #66, NZ_CP155048.1), for which no pyocin-encoding cluster was reported. Following analysis via TattleTail, manual coordinate-level inspection showed that all 5 contained the required *trpE*, *trpG*, *prtN*, *prtR*, holin, and endolysin hits at the whole-genome level. However, in these genomes, the distances between some of the required markers exceed 15,000 bp, the default value of the “window” parameter for TattleTail, preventing the markers from being grouped into a single candidate cluster, resulting in no pyocin gene clusters being found.

For NZ_CP029090.1 (case #10), the genomic distance between the endolysin and *trpG* hits is 31,223 bp. When the “window” parameter was increased to 32,000 bp and the genome was reanalyzed, a pyocin-encoding gene cluster was reported. For NZ_CP021775.1 (case #12), the genomic distance between *trpE* and *prtN* was 45,858 bp. Increasing the “window” parameter to 46,000 bp similarly resulted in a reported pyocin-encoding gene cluster. For NZ_CP194326.1 (case #15) and NZ_CP081202.1 (case #19), the distance between the endolysin and *trpG* hits was 31,272 and 31,265 bp, respectively. In both cases, increasing the “window” parameter to 32,000 bp resulted in a pyocin-encoding gene cluster.

Upon analyzing NZ_CP155048.1 (case #66), the required markers were not grouped into a single cluster under the default 15,000-bp window because the complete marker chain contained a maximum gap of 68,410 bp, resulting in no pyocin-encoding gene cluster being reported. Additionally, when a sufficiently large window was used to reconstruct the potential region, one predicted protein within the region produced qualifying matches to integrase reference proteins and triggered the cluster-level exclusion rule.

The genomic regions between the marker genes, endolysin and *trpG* (for NZ_CP029090.1, NZ_CP194326.1, and NZ_CP081202.1) and *trpE* and *prtN* (for NZ_CP021775.1), possessed several genes that are not associated with canonical pyocin-encoding gene clusters. These genes included, but were not limited to, helix-turn-helix transcriptional regulators, inovirus Gp2 family protein, OB fold protein, AAA family ATPase, RadC family protein recombination directionality factor, and YqaJ viral recombinase family nuclease. These cases illustrate that the default TattleTail parameters may not detect pyocin-related regions with atypical genomic organization or unusually large distances between conserved markers. However, increasing the “window” parameter does not necessarily provide a more accurate prediction. In particular, a larger window may group biologically unrelated genes or mobile-element-associated regions into the same candidate region, as illustrated by case #66. Therefore, regions identified only after increasing the window parameter should be considered noncanonical candidates requiring additional manual evaluation, rather than as confirmed canonical pyocin-encoding gene clusters.

The current iteration of TattleTail is restricted to *P. aeruginosa*, as demonstrated by the analysis of 60 non-*P. aeruginosa Pseudomonas* genomes containing tailocin-encoding gene clusters located between *mutS* and *cinA*, none of which were identified by TattleTail.

In addition, as with other database-driven software, TattleTail may have limited sensitivity for highly divergent or previously uncharacterized pyocins whose sequences are not well represented in existing databases. Furthermore, annotation of gene products may not always achieve complete accuracy, as is common for automated genome annotation tools. TattleTail currently analyzes genome assemblies without reconstructing genomic regions across contig boundaries. Thus, pyocin loci spanning multiple contigs may not be identified automatically and will require manual evaluation of the individual contig outputs. Given these limitations, it is crucial that TattleTail should be considered a prediction tool, and its results should be interpreted accordingly prior to experimental validation. However, for studies and applications requiring precise functional characterization through experimental validation, for example, the production of recombinant proteins from identified pyocin-encoding gene clusters, additional analyses using complementary tools such as BLASTp [40], HHpred [41,42], and AlphaFold [43] are recommended.

## Discussion

A wide range of specialized tools are available for the systematic identification of nonessential, accessory elements in bacterial genomes, including antibiotic resistance genes [44,45], integrons [44], transposable elements and insertion sequences [46], pdif-flanked resistance modules [47], group II introns [48], and prophages [49]. However, despite the increasing recognition of tailocins as important mediators of bacterial competition, no dedicated genome mining tool has been developed for their identification. TattleTail addresses this gap by providing a targeted bioinformatic approach for the identification of pyocin-encoding gene clusters. In its current implementation, the tool integrates the detection of conserved pyocin-associated genes, with the exclusion of candidate regions containing capsid-, terminase-, or integrase-like hits. This design helps distinguish candidate canonical pyocin regions from genomic regions containing phage-associated features, addressing a key limitation in the annotation of phage-derived elements in bacterial genomes.

This capacity is especially advantageous in large-scale genomic studies, where manual inspection and annotation of genomes to identify pyocin-encoding gene clusters is impractical. For example, systematic reanalysis of genomes in the *P. aeruginosa* database using TattleTail may help clarify whether pyocin-encoding regions are underreported and prophage-encoding regions are correspondingly overestimated. When used in parallel with tools specialized for prophage prediction, the overall accuracy of genomic analysis can be improved. Importantly, TattleTail is not intended to replace existing prophage prediction tools, but rather aims to complement them, enabling more accurate and comprehensive annotation when used in combination.

Accurate genome annotation is critical for experimental design, particularly in studies of bacterial competition, horizontal gene transfer, and prophage induction. Experiments designed based on misannotated prophages could lead to misleading conclusions and confound data interpretation. In such contexts, the combined use of TattleTail with established tools for other mobile genetic elements can improve both experimental design and downstream data analysis.

TattleTail is designed to be scalable and accessible, requiring only genome assemblies as input. Its structured output facilitates straightforward interpretation, and batch processing enables analysis of large genome collections at once.

When tested on the curated benchmark dataset, TattleTail accurately identified pyocin-encoding gene clusters in all 98 *P. aeruginosa* genomes while maintaining high specificity against non-*P. aeruginosa* genomes. Additional benchmarking using independently selected genomes and biologically relevant negative controls further supported the predictive performance of the tool. Application to clinical *P. aeruginosa* isolates enabled the experimental induction and validation of representative TattleTail-predicted pyocins, providing experimental support for the biological relevance of the TattleTail predictions.

Several avenues exist for further development. Future versions of TattleTail will include expanded annotation capabilities, including more comprehensive identification of pyocin-associated genes, classification into R- and F-type systems, and subtype-level resolution (e.g., R1, R2, R5, and F8). Implementation of TattleTail as a web-server-based platform is also planned to improve accessibility and usability. In addition, future development will include integrated visualization of pyocin loci as fully annotated gene clusters across large genome datasets.

In the longer term, TattleTail may be extended beyond pyocins to enable identification of tailocins across diverse bacterial taxa [19], particularly in systems where tailocin biology remains poorly understood. Such developments may facilitate the discovery of previously uncharacterized bacterial defense mechanisms, thereby paving the way for novel research in bacterial competition and ecological niche adaptation. Given their specificity and nonreplicative nature, tailocins represent promising candidates for targeted therapeutic applications, particularly in the context of antibiotic resistant infections. Improved identification and annotation of tailocin systems may therefore contribute to the development of new antibacterial strategies.

TattleTail is provided as a publicly available resource for academic use (https://github.com/xthua/TattleTail). In its current implementation, TattleTail provides a targeted approach for identifying canonical pyocin-encoding gene clusters in *P. aeruginosa*. Its application to publicly available *P. aeruginosa* genomes, together with community feedback received, will support continued refinement and expansion of its capabilities.

## Data Availability

TattleTail and usage instructions are available at https://github.com/xthua/TattleTail. The software and source code are available under the following permanent DOI: https://doi.org/10.5281/zenodo.22720514. The BioProject (PRJDB42114) created for this study contains BioSamples SAMD01907650, SAMD01907651, and SAMD01907652, which correspond to *P. aeruginosa* 003, *P. aeruginosa* 005, and *P. aeruginosa* 012, respectively. The raw reads (sra-study) for *P. aeruginosa* 003, 005, and 012 can be accessed via the following accession number: DRP017444. Draft genome assemblies are provided in Supplementary Folder 2 and have been submitted to DDBJ and are currently awaiting accession number designation. Accession numbers will be provided as soon as they are assigned. For the manual determination of pyocin type, representative reference sequences for each pyocin type can be found under these accession numbers: R1 (WHT11591.1) from *P. aeruginosa* S14 [12], R2 (NP_249311.1) from *P. aeruginosa* PAO1 [31], and R5 (DBA05840.1) from *P. aeruginosa* isolate 3-5 [11].

## Supplementary Materials

Supplementary 1 Figs. S1 and S2 Tables S1 to S8 Supplementary Folders 1 to 3 Supplementary Information 1 and 2
