# Supplementary material for: TattleTail: A Rule-Based Tool for Identifying Canonical Pyocin-Encoding Gene Clusters in Pseudomonas aeruginosa
Source: Comput Struct Biotechnol J. 2026 Sep 30;35(1):0230. doi: 10.34133/csbj.0230 (PMC13624779; doi:10.34133/csbj.0230)
Supplement: Supplementary file 1 [file csbj.0230.f1.zip › Supplementary Tables (1-8) and Supplementary Figures (1-2).docx]

**Supplementary Table 1:** List of 98 *P. aeruginosa* genomes with manually identified pyocin-encoding gene clusters prior to analysis via TattleTail. Genomes were acquired from the RefSeq and Genbank databases. Region Length and Region Position were determined using TattleTail.

**Legend for Supplementary Table 1:**

- Black accession numbers: Contain prophages with varying degrees of completeness (intact, questionable, incomplete) which possess genes encountered within pyocin-encoding gene clusters, including tail-associated genes, holins and endolysins (List of genes in the prophages can be found in Supplementary Table 2).

- Green accession numbers: Contain prophages with varying degrees of completeness (intact, questionable, incomplete) which do not possess genes encountered within pyocin-encoding gene clusters, including tail-associated genes, holins and endolysins.

- Cyan accession numbers: Do not contain prophages.

| **#** | **Genbank/Refseq** | **Assembly** | **Region Length** | **Region Position** |
| --- | --- | --- | --- | --- |
| 1 | NC_002516.2 | GCF_000006765.1 | 31.02 Kb | 672,459 - 703,476 |
| 2 | CP000438.1 | GCF_000014625.1 | 29.18 Kb | 685,281 - 714,460 |
| 3 | FM209186.1 | GCF_000026645.1 | 17.13 Kb | 663,604 - 680,732 |
| 4 | CP002496.1 | GCF_000226155.1 | 17.14 Kb | 663,464 - 680,599 |
| 5 | AP012280.1 | GCF_000284555.1 | 17.00 Kb | 5,938,573 - 5,955,576 |
| 6 | DF126593.1 | GCF_000291745.1 | 17.11 Kb | 4,622,486 - 4,639,594 |
| 7 | CP006705.1 | GCF_000468555.2 | 31.02 Kb | 672,336 - 703,353 |
| 8 | CP006728.1 | GCF_000468935.2 | 30.97 Kb | 672,436 - 703,406 |
| 9 | CP006831.1 | GCF_000484495.2 | 31.02 Kb | 672,459 - 703,476 |
| 10 | CP006832.1 | GCF_000484545.2 | 31.02 Kb | 672,459 - 703,476 |
| 11 | CP007224.1 | GCF_000626655.2 | 17.22 Kb | 664,485 - 681,700 |
| 12 | KN050641.1 | GCF_000743405.1 | 17.13 Kb | 4,268,061 - 4,285,191 |
| 13 | CP021380.2 | GCF_000763245.3 | 31.02 Kb | 677,714 - 708,731 |
| 14 | AP014646.1 | GCF_000829255.1 | 17.00 Kb | 667,450 - 684,453 |
| 15 | AP014622.1 | GCF_000829275.1 | 17.00 Kb | 667,447 - 684,450 |
| 16 | CVVB01000099.1 | GCF_001180485.1 | 17.00 Kb | 68,961 - 85,964 |
| 17 | CVXP01000055.1 | GCF_001180545.1 | 17.00 Kb | 68,974 - 85,977 |
| 18 | CVVW01000239.1 | GCF_001180705.1 | 17.00 Kb | 106,158 - 123,161 |
| 19 | CVVY01000482.1 | GCF_001180765.1 | 17.00 Kb | 7,202 - 24,205 |
| 20 | CVVU01000111.1 | GCF_001180785.1 | 17.00 Kb | 18,151 - 35,154 |
| 21 | AP014651.1 | GCF_001547955.1 | 17.00 Kb | 753,655 - 770,659 |
| 22 | AP014839.2 | GCF_00154813  5.1 | 18.24 Kb | 702,042 - 720,278 |
| 23 | LODH01000055.1 | GCF_001560865.1 | 17.14 Kb | 18,206 - 35,341 |
| 24 | LOHI01000016.1 | GCF_001601585.1 | 28.91 Kb | 18,108 - 47,020 |
| 25 | LOHJ01000009.1 | GCF_001601665.1 | 29.26 Kb | 378,943 - 408,202 |
| 26 | LOHH01000012.1 | GCF_001601745.1 | 17.14 Kb | 18,193 - 35,327 |
| 27 | CP014948.1 | GCF_001606045.1 | 29.43 Kb | 663,105 - 692,536 |
| 28 | CP015001.1 | GCF_001792835.1 | 31.02 Kb | 677,765 - 708,782 |
| 29 | CP015002.1 | GCF_001792855.1 | 31.02 Kb | 708,484 - 739,501 |
| 30 | CP015003.1 | GCF_001792875.1 | 31.02 Kb | 677,758 - 708,775 |
| 31 | LVWC01000001.1 | GCF_001802735.1 | 31.02 Kb | 4,101,523 - 4,132,540 |
| 32 | LVXB01000001.1 | GCF_001806505.1 | 31.02 Kb | 677,817 - 708,834 |
| 33 | CP014999 | GCF_001870265.1 | 31.02 Kb | 708,490 - 739,507 |
| 34 | CP013113.1 | GCF_001879525.1 | 17.13 Kb | 695,743 - 712,872 |
| 35 | CP013477.1 | GCF_001900195.1 | 17.53 Kb | 681,690 - 699,216 |
| 36 | CP013478.1 | GCF_001900225.1 | 17.53 Kb | 681,690 - 699,216 |
| 37 | CP013479.1 | GCF_001900265.1 | 17.53 Kb | 681,690 - 699,216 |
| 38 | CP020603.1 | GCF_002085755.1 | 17.00 Kb | 667,406 - 684,409 |
| 39 | PVXJ01000005.1 | GCF_003032395.1 | 17.13 Kb | 50,307 - 67,435 |
| 40 | CP029605.1 | GCF_003433235.1 | 17.00 Kb | 707,622 - 724,625 |
| 41 | CP012901.1 | GCF_003571505.1 | 17.14 Kb | 6,207,033 - 6,224,167 |
| 42 | CP034429.1 | GCF_003957825.1 | 31.02 Kb | 671,976 - 702,993 |
| 43 | CP034430.1 | GCF_004000495.1 | 17.00 Kb | 815,560 - 832,564 |
| 44 | CP037925.1 | GCF_004355125.1 | 17.13 Kb | 664,894 - 682,026 |
| 45 | CP037926.1 | GCF_004355145.1 | 17.13 Kb | 664,827 - 681,959 |
| 46 | CP041354.1 | GCF_006971785.1 | 17.13 Kb | 653,236 - 670,363 |
| 47 | CP043483.1 | GCF_008330985.1 | 17.00 Kb | 710,592 - 727,595 |
| 48 | CP043549.1 | GCF_008370085.1 | 17.00 Kb | 5,614,848 - 5,631,851 |
| 49 | CP045739.1 | GCF_009662315.1 | 17.00 Kb | 759,584 - 776,587 |
| 50 | CP046069.1 | GCF_009676885.1 | 18.89 Kb | 674,281 - 693,168 |
| 51 | CP049161.1 | GCF_011045375.1 | 17.66 Kb | 669,102 - 686,761 |
| 52 | CP051766.1 | GCF_013114915.1 | 17.00 Kb | 6,139,478 - 6,156,482 |
| 53 | CP051768.1 | GCF_013114935.1 | 17.00 Kb | 6,093,905 - 6,110,909 |
| 54 | CP051770.1 | GCF_013114955.1 | 17.00 Kb | 6,130,550 - 6,147,554 |
| 55 | AP024513.1 | GCF_018409365.1 | 29.43 Kb | 698,481 - 727,911 |
| 56 | BSAL01000015.1 | GCF_026012655.1 | 29.16 Kb | 18,374 - 47,530 |
| 57 | BSAN01000013.1 | GCF_026012695.1 | 29.16 Kb | 110,627 - 139,782 |
| 58 | BSAP01000005.1 | GCF_026012735.1 | 29.16 Kb | 238,074 - 267,229 |
| 59 | BSAQ01000003.1 | GCF_026012755.1 | 31.02 Kb | 198,596 - 229,613 |
| 60 | BSAR01000003.1 | GCF_026012775.1 | 31.02 Kb | 244,746 - 275,763 |
| 61 | BSAT01000022.1 | GCF_026012815.1 | 17.00 Kb | 64,525 - 81,529 |
| 62 | BSAU01000004.1 | GCF_026012835.1 | 31.02 Kb | 250,828 - 281,845 |
| 63 | BSAW01000005.1 | GCF_026012875.1 | 17.00 Kb | 18,510 - 35,513 |
| 64 | BSAX01000005.1 | GCF_026012895.1 | 29.26 Kb | 18,403 - 47,662 |
| 65 | BSAZ01000031.1 | GCF_026012935.1 | 17.00 Kb | 43,560 - 60,564 |
| 66 | BSBB01000004.1 | GCF_026012975.1 | 31.02 Kb | 18,316 - 49,333 |
| 67 | BSBC01000003.1 | GCF_026012995.1 | 29.26 Kb | 18,403 - 47,662 |
| 68 | BSBD01000032.1 | GCF_026013015.1 | 16.99 Kb | 42,852 - 59,845 |
| 69 | CP123953.1 | GCF_029916825.1 | 29.13 Kb | 697,412 - 726,541 |
| 70 | CP136598.1 | GCF_032847385.1 | 17.00 Kb | 741,442 - 758,445 |
| 71 | BAABSN010000004.1 | GCF_039544715.1 | 17.00 Kb | 18,377 - 35,376 |
| 72 | CP173019.1 | GCF_044758985.1 | 31.02 Kb | 1,675,859 - 1,706,876 |
| 73 | CP173020.1 | GCF_044758995.1 | 31.02 Kb | 4,180,820 - 4,211,839 |
| 74 | CP173021.1 | GCF_044759005.1 | 17.00 Kb | 670,460 - 687,462 |
| 75 | CP173022.1 | GCF_044759015.1 | 17.00 Kb | 670,460 - 687,462 |
| 76 | CP173023.1 | GCF_044759025.1 | 17.00 Kb | 670,435 - 687,437 |
| 77 | CP069363.1 | GCF_045159815.1 | 17.43 Kb | 674,197 - 691,621 |
| 78 | LN870292.1 | GCF_900069025.1 | 17.53 Kb | 682,172 - 699,698 |
| 79 | LN871187.1 | GCF_900070375.1 | 31.02 Kb | 672,464 - 703,481 |
| 80 | LT608330.1 | GCF_900095805.1 | 29.18 Kb | 685,282 - 714,461 |
| 81 | LT673656.1 | GCF_900149285.1 | 17.49 Kb | 662,390 - 679,877 |
| 82 | LT969520.1 | GCF_900243355.1 | 17.00 Kb | 724,783 - 741,786 |
| 83 | UWWR01000001.1 | GCF_900607915.1 | 18.89 Kb | 989,547 - 1,008,432 |
| 84 | UWXD01000001.1 | GCF_900608035.1 | 17.13 Kb | 636,493 - 653,622 |
| 85 | UWXL01000001.1 | GCF_900608125.1 | 18.89 Kb | 2,778,154 - 2,797,044 |
| 86 | LR130527.1 | GCF_900618255.1 | 17.14 Kb | 3,787,906 - 3,805,041 |
| 87 | LR130534.1 | GCF_900618285.1 | 17.14 Kb | 742,835 - 759,970 |
| 88 | LR130533.1 | GCF_900618305.1 | 17.14 Kb | 4,061,846 - 4,078,981 |
| 89 | LR130536.1 | GCF_900618315.1 | 17.14 Kb | 673,408 - 690,543 |
| 90 | LR130535.1 | GCF_900618325.1 | 17.14 Kb | 673,424 - 690,559 |
| 91 | LR130537.1 | GCF_900618335.1 | 17.14 Kb | 673,395 - 690,530 |
| 92 | OX638701.1 | GCF_951691365.1 | 29.29 Kb | 1,944,236 - 1,973,520 |
| 93 | CATOQB010000003.1 | GCF_951802375.1 | 17.00 Kb | 18,091 - 35,093 |
| 94 | OX638610.1 | GCF_951802375.2 | 17.00 Kb | 18,091 - 35,093 |
| 95 | CATORO010000001.1 | GCF_951805275.1 | 17.00 Kb | 674,279 - 691,281 |
| 96 | OX638564.1 | GCF_951805275.2 | 17.00 Kb | 674,279 - 691,281 |
| 97 | CATODC010000001.1 | GCA_951691365.1 | 29.29 Kb | 1,944,236 - 1,973,520 |
| 98 | LR700248.1 | LR700248.1 | 18.89 Kb | 4,594,995 - 4,613,882 |

**Supplementary Table 2:** The 44 P. aeruginosa genomes (from the 98 *P. aeruginosa* genomes with manually identified pyocin-encoding gene clusters) containing prophages which possess genes encountered within pyocin-encoding gene clusters, including tail-associated genes, holins and endolysins.

| **#** | **Bacterial Strain** | **Region** | **Completeness** | **Score** | **Gene** | **Gene region position** |
| --- | --- | --- | --- | --- | --- | --- |
| 1 | *P. aeruginosa* M18 (CP002496.1) | 2 | intact | 140 | Tail protein | 1484474 – 1486615 |
|  |  |  |  |  | Tail fibers protein | 1,488,836 – 1,489,246 |
|  |  |  |  |  | Holin | 1,473,375 - 1,473,707 |
| 2 | *P. aeruginosa* NCGM2.S1 (AP012280.1) | 1 | intact | 150 | Minor tail protein L | 1,731,951 – 1,732,697 |
|  |  |  |  |  | Minor tail protein K | 1,732,700 – 1,733,458 |
|  |  |  |  |  | Tail assembly protein | 1,734,209 – 1,734,781 |
|  |  |  |  |  | Phage tail protein | 1,734,838 - 1,738,290 |
|  |  |  |  |  | Phage tail protein | 1,739,251 – 1,740,375 |
|  |  | 3 | intact | 150 | contractile tail sheath protein | 4,198,543 – 4,199,703 |
|  |  |  |  |  | Tail protein I | 4,202,286 – 4,202,816 |
|  |  | 4 | intact | 140 | Putative holin protein | 5,280,837 – 5,281,133 |
| 3 | *P. aeruginosa* NCMG1179 (DF126593.1) | 1 | intact | 100 | Tail fibers protein | 739,918 – 740,328 |
|  |  |  |  |  | Holin | 722,459 – 722,791 |
|  |  | 3 | intact | 100 | Minor tail protein M | 3,724,270 – 3,724,608 |
|  |  |  |  |  | Minor tail protein L | 3,723,524 – 3,724,273 |
|  |  |  |  |  | Minor tail protein | 3,722,760 – 3,723,521 |
|  |  |  |  |  | Central tail fiber | 3,715,145 – 3,718,807 |
| 4 | *P. aeruginosa* strain PAG (KN050641.1) | 1 | intact | 150 | Tail protein | 1,862,799 – 1,863,335 |
|  |  |  |  |  | Tail protein I | 1,865,680 – 1,866,216 |
|  |  |  |  |  | Tail sheath protein | 1,869,212 – 1,870,387 |
|  |  |  |  |  | Major tail tube protein | 1,870,444 – 1,870,959 |
|  |  |  |  |  | Tail protein | 1,874,226 – 1,874,666 |
|  |  |  |  |  | Tail protein | 1,874,663 – 1,875,937 |
|  |  | 5 | Intact | 100 | Tail fibers protein | 4,962,841 – 4,963,251 |
|  |  |  |  |  | Holin | 4,945,382 – 4,945,714 |
| 5 | *P. aeruginosa* strain CCBH4851 (CP021380.2) | 3 | intact | 150 | Tail fiber | 2,765,206 – 2,765,760 |
|  |  |  |  |  | Tail protein | 2,767,826 – 2,768,932 |
|  |  |  |  |  | Phage major tail tube protein | 2,774,180 – 2,774,704 |
|  |  |  |  |  | Major tail sheath protein | 2,774,705 – 2,776,132 |
|  |  | 4 | intact | 150 | Tail protein | 2,840,013 – 2,842,760 |
|  |  | 6 | intact | 140 | Central fiber J | 4,925,642 – 4,929,307 |
|  |  |  |  |  | Minor tail protein K | 4,930,498 – 4,931,256 |
|  |  |  |  |  | Minor tail protein L | 4,931,259 – 4,932,005 |
| 6 | *P. aeruginosa* NCGM 1984 (AP014646.1) | 2 | intact | 150 | Tail protein | 1336799..1339006 |
|  |  | 4 | questionable | 70 | Tail protein | 3710023..3711147 |
|  |  |  |  |  | Central tail fiber | 3712107..3715718 |
|  |  |  |  |  | Phage minor tail protein L | 3717134..3717835 |
|  |  | 6 | intact | 150 | Tail protein | 4354358..4356565 |
|  |  | 8 | Intact | 150 | Phage tail protein | 4978356..4979480 |
|  |  |  |  |  | Central tail fiber J | 4980440..4984009 |
|  |  |  |  |  | Minor tail protein K | 4985389..4986147 |
|  |  |  |  |  | Minor tail protein L | 4986150..4986896 |
|  |  | 9 | intact | 150 | Tail protein | 5639361..5641568 |
|  |  | 10 | intact | 150 | tail protein I | 6296267..6296884 |
|  |  |  |  |  | Tail fiber protein | 6296881..6298698 |
|  |  |  |  |  | tail protein | 6298708..6299142 |
|  |  |  |  |  | tail sheath protein | 6299243..6300418 |
|  |  |  |  |  | major tail tube protein | 6300475..6300990 |
|  |  |  |  |  | Tail protein | 6304256..6304696 |
|  |  |  |  |  | Tail protein | 6304693..6305970 |
|  |  |  |  |  | Holin family protein | 6291775..6292047 |
| 7 | *P. aeruginosa* NCGM 1900 (AP014622.1) | 2 | intact | 150 | Tail protein | 1336794..1339001 |
|  |  | 4 | intact | 150 | Phage tail protein | 1960789..1961913 |
|  |  |  |  |  | Central tail fiber J | 1962873..1966442 |
|  |  |  |  |  | Minor tail protein K | 1967822..1968580 |
|  |  |  |  |  | Minor tail protein L | 1968583..1969329 |
|  |  | 5 | intact | 150 | Tail protein | 2621792..2623999 |
|  |  | 7 | questionable | 70 | Phage tail protein | 4995011..4996135 |
|  |  |  |  |  | Central tail fiber | 4997095..5000706 |
|  |  |  |  |  | Phage tail minor protein L | 5002122..5002823 |
|  |  | 9 | intact | 150 | Tail protein | 5639345..5641552 |
| 8 | *P. aeruginosa* NCGM257 (AP014651.1) | 1 | intact | 150 | Tail protein | complement(493211..495418) |
|  |  | 4 | intact | 150 | Tail protein I | 1623945..1624475 |
|  |  |  |  |  | Tail sheath protein | 1627058..1628218 |
|  |  | 5 | intact | 150 | Tail protein | 2674834..2675487 |
|  |  | 6 | incomplete | 60 | Central tail fiber | 2687695..2691261 |
|  |  |  |  |  | Phage tail protein | 2692221..2693345 |
|  |  | 7 | intact | 150 | Tail protein | complement(3111201..3113408) |
|  |  | 8 | intact | 150 | Phage tail protein | complement(5086868..5087992) |
|  |  |  |  |  | Central tail fiber | complement(5088952..5092443) |
|  |  | 12 | intact | 150 | Holin family protein | 6571960..6572232 |
|  |  |  |  |  | Tail protein I | 6576452..6577069 |
|  |  |  |  |  | Tail fiber protein | 6577066..6578883 |
| 9 | *P. aeruginosa* strain 8380 (AP014839.2) | 3 | intact | 150 | Tail protein I | 1518846..1519376 |
|  |  |  |  |  | Tail sheath protein | 1522226..1523386 |
| 10 | *P. aeruginosa* strain N17-1 (CP014948.1) | 2 | intact | 130 | glycoside hydrolase family 19 | 2634980..2635597 |
| 11 | *P. aeruginosa* strain PA1088 (CP015001.1) | 3 | intact | 140 | Central tail fiber J | complement(4777610..4781275) |
|  |  |  |  |  | Minor tail protein K | complement(4782466..4783224) |
|  |  |  |  |  | Minor tail protein L | complement(4783227..4783973) |
|  |  | 5 | intact | 150 | Gp25 tail fiber | complement(5491338..5491892) |
|  |  |  |  |  | Tail protein | complement(5493958..5495064) |
|  |  |  |  |  | Gp14 phage major tail tube protein | complement(5500312..5500836) |
|  |  |  |  |  | major tail sheath protein | complement(5500837..5502264) |
| 12 | *P. aeruginosa* strain PA8281 (CP015002.1) | 1 | intact | 150 | Tail fiber protein | complement(640648..643269) |
|  |  |  |  |  | Tail protein | complement(643279..643803) |
|  |  | 4 | intact | 150 | Tail protein | 2832559..2835306 |
|  |  | 5 | intact | 150 | Major tail sheath protein | 3344149..3345576 |
|  |  |  |  |  | Gp14 Phage major tail tube protein | 3345577..3346101 |
|  |  |  |  |  | Tail protein | 3351349..3352455 |
|  |  |  |  |  | Gp25 tail fiber | 3354521..3355075 |
|  |  | 7 | intact | 150 | Putative holin protein | 5638398..5638694 |
| 13 | *P. aeruginosa* strain PA11803 (CP015003.1) | 2 | Intact | 150 | Tail fibers protein | 927493..927903 |
|  |  |  |  |  | Tail fibers protein | 968687..969097 |
|  |  | 3 | intact | 150 | Tail fibers protein | 1353092..1353502 |
|  |  |  |  |  | Tail fibers protein | 1395395..1395805 |
|  |  | 5 | intact | 150 | Major tail sheath protein | 3424676..3426103 |
|  |  |  |  |  | Gp14 phage major tail tube protein | 3426104..3426628 |
|  |  |  |  |  | Tail protein | 3431876..3432982 |
|  |  |  |  |  | Gp25 tail fiber | 3435048..3435602 |
|  |  | 8 | intact | 140 | Central tail fiber J | complement(5074107..5077772) |
|  |  |  |  |  | Minor tail protein K | complement(5078963..5079721) |
|  |  |  |  |  | Minor tail protein L | complement(5079724..5080470) |
|  |  | 10 | intact | 150 | Putative holin protein | complement(5920695..5920991) |
| 14 | *P. aeruginosa* strain PA3448  (LVWC01000001.1) | 1 | intact | 140 | Central tail fiber J | complement(1486074..1489739) |
|  |  |  |  |  | Minor tail protein K | complement(1490930..1491688) |
|  |  |  |  |  | Minor tail protein L | complement(1491691..1492437) |
|  |  | 3 | intact | 150 | Tail fiber protein | complement(4033687..4036308) |
|  |  |  |  |  | Tail protein | complement(4036318..4036842) |
|  |  | 5 | intact | 150 | Gp25 tail fiber | complement(4472568..4473122) |
|  |  |  |  |  | Tail protein | complement(4475188..4476294) |
|  |  |  |  |  | Gp14 phage major tail tube protein | complement(4481542..4482066) |
|  |  |  |  |  | Major tail sheath protein | complement(4482067..4483494) |
| 15 | *P. aeruginosa* strain PA12117  (LVXB01000001.1) | 2 | intact | 150 | Gp25 tail fiber | complement(1048863..1049417) |
|  |  |  |  |  | Tail protein | complement(1051483..1052589) |
|  |  |  |  |  | Gp14 phage major tail tube protein | complement(1057837..1058361) |
|  |  |  |  |  | Major tail sheath protein | complement(1058362..1059789) |
| 16 | *P. aeruginosa* strain PA7790 (CP014999) | 1 | intact | 150 | Tail fiber protein | complement(640654..643275) |
|  |  |  |  |  | Tail protein | complement(643285..643809) |
|  |  | 4 | intact | 150 | Tail protein | 2827703..2830450 |
|  |  | 5 | intact | 150 | Major tail sheath protein | 3325387..3326814 |
|  |  |  |  |  | Gp14 phage major tail tube protein | 3326815..3327339 |
|  |  |  |  |  | Tail protein | 3332587..3333693 |
|  |  |  |  |  | Gp25 tail fiber | 3335759..3336313 |
|  |  | 9 | intact | 150 | Putative holin protein | 5701665..5701961 |
| 17 | *P. aeruginosa* strain PAER4_119  (CP013113.1) | 2 | intact | 150 | Tail protein | complement(2588176..2589447) |
|  |  |  |  |  | Tail protein | complement(2589444..2589884) |
|  |  |  |  |  | Major tail tube protein | complement(2593159..2593674) |
|  |  |  |  |  | Tail sheath protein | complement(2593731..2594906) |
|  |  |  |  |  | Tail protein I | complement(2597902..2598438) |
|  |  |  |  |  | Tail protein | complement(2601329..2601865) |
|  |  |  |  |  | Predicted lysis | complement(2601943..2602404) |
|  |  |  |  |  | Major holin family protein | complement(2603204..2603476) |
| 18 | *P. aeruginosa* strain E6130952  (CP020603.1) | 2 | intact | 150 | Glycoside hydrolase family 19 | 880298..880909 |
|  |  |  |  |  | minor tail protein L | 894502..895248 |
|  |  |  |  |  | minor tail protein K | 895251..896009 |
|  |  |  |  |  | Phage tail protein | 897120..900572 |
|  |  |  |  |  | Phage tail protein | 901532..902656 |
|  |  | 3 | intact | 150 | Tail protein | 1377232..1379439 |
|  |  | 7 | intact | 150 | Phage tail protein | complement(3896319..3897443) |
|  |  |  |  |  | Central tail fiber | complement(3898403..3901894) |
|  |  |  |  |  | Gp18, phage minor tail protein L | complement(3903310..3904011) |
|  |  | 8 | intact | 150 | Tail protein | 4535709..4537916 |
|  |  | 9 | intact | 150 | Phage tail protein | complement(4831723..4832847) |
|  |  |  |  |  | Central tail fiber | complement(4833807..4837298) |
|  |  |  |  |  | Gp18, phage minor tail protein L | complement(4838714..4839415) |
|  |  | 10 | intact | 150 | Phage tail protein | complement(5157342..5158466) |
|  |  |  |  |  | Phage tail protein | complement(5159427..5162879) |
|  |  |  |  |  | Minor tail protein K | complement(5164259..5165017) |
|  |  |  |  |  | Minor tail protein L | complement(5165020..5165766) |
|  |  |  |  |  | Glycoside hydrolase family 19 | complement(5179305..5179916) |
|  |  |  |  |  |  |  |
| 19 | *P. aeruginosa* strain 24Pae112  (CP029605.1) | 6 | intact | 150 | Phage tail protein | complement(3881888..3883012) |
|  |  |  |  |  | Central tail fiber | complement(3883972..3887463) |
|  |  |  |  |  | Gp18 phage minor tail protein L | complement(3888879..3889580) |
|  |  | 7 | intact | 150 | Phage tail protein | complement(5053885..5055009) |
|  |  |  |  |  | Central tail fiber J | complement(5055969..5059535) |
|  |  |  |  |  | Minor tail protein K | complement(5061547..5062305) |
|  |  |  |  |  | Minor tail protein L | complement(5062308..5063054) |
|  |  |  |  |  | Glycoside hydrolase family 19 | complement(5076592..5077203) |
|  |  | 10 | intact | 150 | Tail protein | 6604840..6605376 |
|  |  |  |  |  | Tail protein I | 6608504..6609121 |
|  |  |  |  |  | Tail fiber protein | 6609118..6610935 |
|  |  |  |  |  | Tail protein | 6610945..6611379 |
|  |  |  |  |  | Tail sheath protein | 6611480..6612655 |
|  |  |  |  |  | Major tail tube protein | 6612712..6613227 |
|  |  |  |  |  | Tail protein | 6616494..6616934 |
|  |  |  |  |  | Tail protein | 6616931..6618208 |
| 20 | *P. aeruginosa* strain N15-01092 (CP012901.1) | 2 | intact | 150 | Tail protein I | 1922048..1922578 |
|  |  |  |  |  | Tail sheath protein | 1925161..1926321 |
|  |  | 3 | intact | 150 | Major tail protein | 2410007..2410795 |
|  |  | 4 | intact | 130 | holin | 3004937..3005194 |
|  |  | 5 | questionable | 80 | Minor tail protein K | complement(4344651..4345445) |
|  |  | 6 | intact | 120 | Tail fibers protein | complement(5592255..5592665) |
|  |  |  |  |  | Tail protein | complement(5594886..5597027) |
|  |  |  |  |  | Glycoside hydrolase family 19 | complement(5607246..5607863) |
|  |  | 7 | intact | 150 | Phage tail protein | complement(5983529..5984503) |
|  |  |  |  |  | Central tail fiber J | complement(5986661..5990224) |
|  |  |  |  |  | Minor tail protein K | complement(5991169..5991927) |
|  |  |  |  |  | Minor tail protein L | complement(5991930..5992676) |
|  |  |  |  |  | Glycoside hydrolase family 19 | complement(6006271..6006882) |
| 21 | *P. aeruginosa* strain GIMC5016:PA1840 (CP034430.1) | 3 | intact | 136 | Tail protein | 1263223..1265430 |
| 22 | *P. aeruginosa* strain AES1M  (CP037925.1) | 3 | intact | 150 | Gp25, Tail fiber | complement(2095947..2096501) |
|  |  |  |  |  | Tail protein | complement(2098567..2099661) |
|  |  |  |  |  | Gp14, Phage major tail tube protein | complement(2104456..2104980) |
|  |  |  |  |  | Major tail sheath protein | complement(2104981..2106408) |
|  |  | 4 | intact | 130 | Glycoside hydrolase family 19 | complement(3421640..3422257) |
| 23 | *P. aeruginosa* strain AES1R  (CP037926.1) | 3 | intact | 130 | Glycoside hydrolase family 19 | 2655374..2655991 |
|  |  | 4 | intact | 150 | Major tail sheath protein | 3970996..3972423 |
|  |  |  |  |  | Gp14, Phage major tail tube protein | 3972424..3972948 |
|  |  |  |  |  | Tail protein | 3977743..3978837 |
|  |  |  |  |  | Gp25, tail fiber | 3980903..3981457 |
| 24 | *P. aeruginosa* strain GIMC5001:PAT-23  (CP043483.1) | 4 | intact | 150 | Phage tail protein | complement(4526035..4527159) |
|  |  |  |  |  | Phage tail protein | complement(4528120..4531572) |
|  |  |  |  |  | Minor tail protein K | complement(4532952..4533710) |
|  |  |  |  |  | Minor tail protein L | complement(4533713..4534459) |
|  |  |  |  |  | Glycoside hydrolase family 19 | complement(4547998..4548609) |
| 25 | *P. aeruginosa* strain GIMC5002:PAT-169 (CP043549.1) | 1 | intact | 150 | Glycoside hydrolase family 19 | 1745618..1746229 |
|  |  |  |  |  | Minor tail protein L | 1759768..1760514 |
|  |  |  |  |  | Minor tail protein K | 1760517..1761275 |
|  |  |  |  |  | Phage tail protein | 1762655..1766107 |
|  |  |  |  |  | Phage tail protein | 1767068..1768192 |
| 26 | *P. aeruginosa* strain AG1  (CP045739.1) | 2 | intact | 100 | Glycoside hydrolase family 19 | 975824..976441 |
|  |  |  |  |  | Tail fibers protein | 992939..993349 |
|  |  | 3 | intact | 110 | Tail protein | 4721198..4723405 |
|  |  | 5 | intact | 150 | Tail protein | 5688294..5688818 |
|  |  |  |  |  | Tail fiber protein | 5688830..5690794 |
| 27 | *P. aeruginosa* strain KRP1  (CP046069.1) | 2 | intact | 150 | Holin family protein | 4874250..4874522 |
|  |  |  |  |  | Tail protein | 4876100..4876636 |
|  |  |  |  |  | Tail protein I | 4878981..4879517 |
|  |  |  |  |  | Tail sheath protein | 4882513..4883688 |
|  |  |  |  |  | Major tail tube protein | 4883745..4884260 |
|  |  |  |  |  | Tail protein | 4887527..4887967 |
|  |  |  |  |  | Tail protein | 4887964..4889247 |
|  |  | 4 | intact | 150 | Holin family protein | 6246146..6246418 |
|  |  |  |  |  | Tail protein | 6247996..6248532 |
|  |  |  |  |  | Tail protein I | 6251423..6251959 |
|  |  |  |  |  | Tail sheath protein | 6254870..6256045 |
|  |  |  |  |  | Major tail tube proein | 6256102..6256617 |
|  |  |  |  |  | Tail protein | 6259872..6260312 |
|  |  |  |  |  | Tail protein | 6260309..6261595 |
| 28 | *P. aeruginosa* Pa12 (AP024513.1) | 1 | intact | 150 | Tail fiber protein | complement(630742..633375) |
|  |  |  |  |  | Tail protein | complement(633385..633909) |
| 29 | *P. aeruginosa* strain 59  (CP123953.1) | 1 | intact | 150 | Tail fiber protein | complement(629577..632198) |
|  |  |  |  |  | Tail protein | complement(632208..632732) |
|  |  | 3 | intact | 130 | Holin | 1271000..1271314 |
|  |  |  |  |  | Glycoside hydrolase family 19 | 1271314..1271931 |
|  |  |  |  |  | Tail fibers protein | 1288811..1289221 |
|  |  | 7 | intact | 120 | holin | complement(5078159..5078416) |
|  |  | 9 | intact | 150 | Holin family protein | 6812444..6812716 |
|  |  |  |  |  | Tail protein | 6814055..6814591 |
|  |  |  |  |  | Tail protein I | 6816936..6817472 |
|  |  |  |  |  | Major tail sheath protein | 6820368..6821543 |
|  |  |  |  |  | Major tail tube protein | 6821600..6822115 |
|  |  |  |  |  | Tail protein | 6825389..6825829 |
|  |  |  |  |  | Tail protein | 6825826..6827103 |
| 30 | *P. aeruginosa* strain PA12  (CP136598.1) | 1 | intact | 150 | Tail fiber protein | complement(674730..676694) |
|  |  |  |  |  | Tail protein | complement(676706..677230) |
|  |  | 7 | intact | 150 | Holin family protein | 5234490..5234762 |
|  |  |  |  |  | Tail protein I | 5238985..5239602 |
|  |  |  |  |  | Tail fiber protein | 5239599..5241416 |
|  |  |  |  |  | Tail protein | 5241426..5241860 |
|  |  |  |  |  | Major tail sheath protein | 5241961..5243136 |
|  |  |  |  |  | Major tail tube protein | 5243193..5243708 |
|  |  |  |  |  | Tail protein | 5246930..5247370 |
|  |  |  |  |  | Tail protein | 5247367..5248662 |
| 31 | *P. aeruginosa* strain PA6567 (CP173021.1) | 2 | intact | 110 | Glycoside hydrolase family 19 | 892808..893425 |
|  |  |  |  |  | Tail protein | 903634..905775 |
|  |  |  |  |  | Tail fibers protein | 907996..908406 |
|  |  | 4 | intact | 120 | holin | 1445737..1446051 |
|  |  |  |  |  | Glycoside hydrolase family 19 | 1446051..1446668 |
|  |  |  |  |  | Tail fibers protein | 1463548..1463958 |
|  |  | 6 | intact | 150 | Phage tail protein | complement(4180026..4181000) |
|  |  |  |  |  | Central tail fiber | complement(4181964..4185569) |
|  |  |  |  |  | Gp18, Phage minor tail protein L | complement(4186985..4187686) |
| 32 | *P. aeruginosa* strain PA6536  (CP173022.1) | 2 | intact | 110 | Glycoside hydrolase family 19 | 892808..893425 |
|  |  |  |  |  | Tail protein | 903634..905775 |
|  |  |  |  |  | Tail fibers protein | 907996..908406 |
|  |  | 4 | intact | 120 | holin | 1381798..1382112 |
|  |  |  |  |  | Glycoside hydrolase family 19 | 1382112..1382729 |
|  |  |  |  |  | Tail fibers protein | 1399609..1400019 |
|  |  | 6 | questionable | 70 | Phage tail protein | complement(4023632..4024606) |
|  |  |  |  |  | Central tail fiber | complement(4025570..4029175) |
|  |  |  |  |  | Phage tail assembly protein | complement(4029806..4030588) |
|  |  |  |  |  | Gp18, Phage minor tail protein L | complement(4030591..4031292) |
| 33 | *P. aeruginosa* strain PA5957  (CP173023.1) | 2 | intact | 110 | Glycoside hydrolase family 19 | 892783..893400 |
|  |  |  |  |  | Tail protein | 903609..905750 |
|  |  |  |  |  | Tail fibers protein | 907971..908381 |
|  |  | 4 | intact | 120 | holin | 1381772..1382086 |
|  |  |  |  |  | Glycoside hydrolase family 19 | 1382086..1382703 |
|  |  |  |  |  | Tail fibers protein | 1399583..1399993 |
| 34 | *P. aeruginosa* isolate RW109  (LT969520.1) | 4 | intact | 107 | Glycoside hydrolase family 19 | 1395957..1396574 |
|  |  |  |  |  | Tail fibers protein | 1412298..1412708 |
| 35 | *P. aeruginosa* paerg006  (UWWR01000001.1) | 1 | intact | 150 | Holin family protein | 699872..700144 |
|  |  |  |  |  | Tail protein | 701722..702258 |
|  |  |  |  |  | Tail protein I | 705149..705685 |
|  |  |  |  |  | Tail sheath protein | 708596..709771 |
|  |  |  |  |  | Major tail tube protein | 709828..710340 |
|  |  |  |  |  | Tail protein | 713595..714035 |
|  |  |  |  |  | Tail protein | 714032..715318 |
| 36 | *P. aeruginosa* isolate paerg007 (UWXD01000001.1) | 4 | intact | 150 | Tail fiber protein | complement(4237510..4240239) |
|  |  | 7 | intact | 150 | Tail protein | 5816419..5816946 |
|  |  |  |  |  | Tail protein I | 5819286..5819903 |
|  |  |  |  |  | Tail fiber protein | 5819900..5821516 |
|  |  |  |  |  | Tail sheath protein | 5822036..5823208 |
|  |  |  |  |  | Major tail tube protein | 5823257..5823772 |
|  |  |  |  |  | Tail protein | 5826890..5827333 |
|  |  |  |  |  | Tail protein | 5827330..5828589 |
| 37 | *P. aeruginosa* isolate paerg008  (UWXL01000001.1) | 1 | intact | 150 | Holin family protein | 1616984..1617256 |
|  |  |  |  |  | Tail protein | 1618834..1619370 |
|  |  |  |  |  | Tail protein I | 1622260..1622796 |
|  |  |  |  |  | Tail sheath protein | 1625707..1626882 |
|  |  |  |  |  | Major tail tube protein | 1626939..1627454 |
|  |  |  |  |  | Tail protein | 1630709..1631149 |
|  |  |  |  |  | Tail protein | 1631146..1632432 |
| 38 | *P. aeruginosa* isolate paerg005  (LR130534.1) | 2 | intact | 110 | holin | 1489690..1490022 |
|  |  |  |  |  | Glycoside hydrolase family 19 | 1490019..1490636 |
|  |  |  |  |  | Tail fibers protein | 1506360..1506770 |
|  |  | 5 | intact | 130 | Tail fibers protein | complement(5200572..5200982) |
|  |  |  |  |  | Glycoside hydrolase family 19 | complement(5217027..5217644) |
| 39 | *P. aeruginosa* isolate paerg009 (LR130533.1) | 2 | intact | 130 | Tail fibers protein | complement(1591648..1592058) |
|  |  |  |  |  | Glycoside hydrolase family 19 | complement(1608101..1608718) |
|  |  | 4 | intact | 110 | holin | 4806255..4806587 |
|  |  |  |  |  | Glycoside hydrolase family 19 | 4806584..4807201 |
|  |  |  |  |  | Tail fibers protein | 4822922..4823332 |
| 40 | *P. aeruginosa* strain 4782MK  (OX638701.1) | 4 | intact | 150 | Tail protein | 6937171..6937695 |
|  |  |  |  |  | Tail fiber protein | 6937707..6939671 |
| 41 | *P. aeruginosa* strain 3796A  (CATORO010000001.1) | 3 | questionable | 70 | Phage tail protein | complement(4002784..4003758) |
|  |  |  |  |  | Central tail fiber | complement(4004722..4008327) |
|  |  |  |  |  | Gp18, phage minor tail protein L | complement(4009743..4010444) |
| 42 | *P. aeruginosa* strain 3796A  (OX638564.1) | 3 | Questionable | 70 | Phage tail protein | complement(4002784..4003758) |
|  |  |  |  |  | Central tail fiber | complement(4004722..4008327) |
|  |  |  |  |  | Gp18, phage minor tail protein L | complement(4009743..4010444) |
| 43 | *P. aeruginosa* strain 4782MK  (CATODC010000001.1) | 4 | intact | 150 | Tail protein | 6937171..6937695 |
|  |  |  |  |  | Tail fiber protein | 6937707..6939671 |
| 44 | *P. aeruginosa* isolate ID40  (LR700248.1) | 4 | intact | 150 | Tail protein | complement(5028524..5030731) |
|  |  | 5 | intact | 150 | Tail protein | 6549632..6550285 |
|  |  |  |  |  | Minor tail protein M | 6554135..6554473 |
|  |  |  |  |  | Minor tail protein L | 6554470..6555219 |
|  |  |  |  |  | Minor tail protein | 6555222..6555977 |
|  |  |  |  |  | Central J fiber | 6562475..6566137 |

**Supplementary Table 3:** 100 independently, randomly selected *P. aeruginosa* genomes for analysis via TattleTail. Region Length and Region Position were determined using TattleTail.

| **#** | **Genbank/Refseq** | **Assembly** | **Region Length** | **Region Position** |
| --- | --- | --- | --- | --- |
| 1 | NZ_LN831024.1 | GCA_001457615.1 | 17.00 Kb | 660,824 - 677,823 |
| 2 | NZ_CP011317.1 | GCA_000981825.1 | 17.00 Kb | 759,045 - 776,048 |
| 3 | NZ_CP138326.1 | GCA_033615795.1 | 18.89 Kb | 6,445,127 - 6,464,015 |
| 4 | NZ_CP097560.1 | GCA_026727755.1 | 17.13 Kb | 733,224 - 750,353 |
| 5 | NZ_CM150494.1 | GCA_056324665.1 | 29.26 Kb | 4,642,371 - 4,671,630 |
| 6 | NZ_CP123953.1 | GCA_029916825.1 | 29.13 Kb | 697,412 - 726,541 |
| 7 | NZ_CP124632.1 | GCA_029961325.1 | 17.13 Kb | 1,085,223 - 1,102,352 |
| 8 | NZ_CM165583.1 | GCA_056782815.1 | 17.00 Kb | 680,359 - 697,362 |
| 9 | NZ_CP008857.1 | GCA_001516265.1 | 17.00 Kb | 5,347,957 - 5,364,960 |
| 10 | NZ_CP029090.1 | GCA_003073795.1 | - | - |
| 11 | NZ_CP150882.1 | GCA_039908025.1 | 17.00 Kb | 713,255 - 730,258 |
| 12 | NZ_CP021775.1 | GCA_002192495.1 | - | - |
| 13 | NZ_CM166498.1 | GCA_056858535.1 | 17.00 Kb | 6,531,517 - 6,548,520 |
| 14 | NZ_CP173021.1 | GCA_044759005.1 | 17.00 Kb | 670,460 - 687,462 |
| 15 | NZ_CP194326.1 | GCA_050908775.1 | - | - |
| 16 | NZ_LS998783.1 | GCA_900497025.1 | 17.00 Kb | 769,197 - 786,200 |
| 17 | NZ_CP169522.1 | GCA_041951335.1 | 17.13 Kb | 4,800,547 - 4,817,676 |
| 18 | NZ_CP199797.1 | GCA_052695735.1 | 17.13 Kb | 895,248 - 912,378 |
| 19 | NZ_CP081202.1 | GCA_019710495.1 | - | - |
| 20 | NZ_CP124646.1 | GCA_029961525.1 | 29.18 Kb | 692,661 - 721,840 |
| 21 | NZ_CP082823.1 | GCA_019915465.1 | 29.20 Kb | 676,321 - 705,520 |
| 22 | NZ_CP191360.1 | GCA_050499725.1 | 29.16 Kb | 676,700 - 705,863 |
| 23 | NZ_CP140515.1 | GCA_034120925.2 | 17.14 Kb | 800,527 - 817,662 |
| 24 | NZ_CP124660.1 | GCA_029962185.1 | 17.24 Kb | 723,282 - 740,525 |
| 25 | NZ_CP199209.1 | GCA_054130125.1 | 17.11 Kb | 677,713 - 694,822 |
| 26 | NZ_CM149887.1 | GCA_056270745.1 | 17.00 Kb | 6,371,361 - 6,388,364 |
| 27 | NZ_CP199218.1 | GCA_054130165.1 | 17.13 Kb | 719,393 - 736,524 |
| 28 | NZ_CP029097.1 | GCA_003073895.1 | 17.13 Kb | 6,554,642 - 6,571,772 |
| 29 | NZ_CP124649.1 | GCA_029961565.1 | 17.14 Kb | 742,743 - 759,878 |
| 30 | NZ_CP082821.1 | GCA_019915485.1 | 29.20 Kb | 676,324 - 705,523 |
| 31 | NZ_OZ483313.1 | GCA_984919515.1 | 29.29 Kb | 2,586,517 - 2,615,801 |
| 32 | NZ_CP086016.1 | GCA_020771755.1 | 29.26 Kb | 735,731 - 764,990 |
| 33 | NZ_OZ484744.1 | GCA_985708255.1 | 18.30 Kb | 6,398,635 - 6,416,939 |
| 34 | NZ_CP116721.1 | GCA_028403985.1 | 17.00 Kb | 755,135 - 772,138 |
| 35 | NZ_CP059995.1 | GCA_014155905.1 | 29.26 Kb | 735,372 - 764,631 |
| 36 | NZ_OX638701.1 | GCA_951691365.2 | 29.29 Kb | 1,944,236 - 1,973,520 |
| 37 | NZ_CP097557.1 | GCA_026727735.1 | 31.05 Kb | 663,175 - 694,228 |
| 38 | NZ_CP033684.1 | GCA_003798105.1 | 18.89 Kb | 719,009 - 737,897 |
| 39 | NZ_CP115481.1 | GCA_036237095.1 | 31.04 Kb | 2,536,571 - 2,567,612 |
| 40 | NZ_CP115235.1 | GCA_036232765.1 | 17.13 Kb | 2,006,624 - 2,023,754 |
| 41 | NZ_CM183662.1 | GCA_059547795.1 | 17.14 Kb | 801,441 - 818,576 |
| 42 | NZ_CM142193.1 | GCA_054881555.1 | 17.14 Kb | 801,441 - 818,576 |
| 43 | NZ_CP116725.1 | GCA_028404105.1 | 17.00 Kb | 732,881 - 749,884 |
| 44 | NZ_CP137491.1 | GCA_033219955.1 | 17.00 Kb | 1,039,290 - 1,056,293 |
| 45 | NZ_CP150132.1 | GCA_032843885.3 | 17.00 Kb | 710,352 - 727,355 |
| 46 | NZ_CP120881.1 | GCA_031224255.1 | 29.26 Kb | 707,099 - 736,358 |
| 47 | NZ_CM148590.1 | GCA_056114335.1 | 17.01 Kb | 1,413,659 - 1,430,664 |
| 48 | NZ_CP087673.1 | GCA_020990445.1 | 17.00 Kb | 668,657 - 685,660 |
| 49 | NZ_CP097709.1 | GCA_024266915.1 | 17.14 Kb | 695,817 - 712,951 |
| 50 | NZ_CP141683.1 | GCA_035621255.1 | 17.14 Kb | 5,870,621 - 5,887,756 |
| 51 | NZ_CP020603.1 | GCA_002085755.1 | 17.00 Kb | 667,406 - 684,409 |
| 52 | NZ_AP040358.1 | GCA_053343655.1 | 17.13 Kb | 680,526 - 697,656 |
| 53 | NZ_CP139036.1 | GCA_042666995.1 | 17.00 Kb | 684,431 - 701,435 |
| 54 | NZ_CP120708.1 | GCA_031220265.1 | 16.98 Kb | 749,078 - 766,056 |
| 55 | NZ_CP014999.1 | GCA_001870265.1 | 31.02 Kb | 708,490 - 739,507 |
| 56 | NZ_CM176358.1 | GCA_057962675.1 | 17.00 Kb | 4,039,018 - 4,056,022 |
| 57 | NZ_CP106742.1 | GCA_027570595.1 | 17.13 Kb | 707,900 - 725,028 |
| 58 | NZ_CP199843.1 | GCA_052697495.1 | 17.13 Kb | 680,945 - 698,075 |
| 59 | NZ_CP043328.1 | GCA_008195485.1 | 17.00 Kb | 676,118 - 693,121 |
| 60 | NZ_CP173126.1 | GCA_051015905.1 | 31.04 Kb | 700,836 - 731,877 |
| 61 | NZ_CP093032.1 | GCA_022570475.1 | 29.18 Kb | 684,824 - 714,003 |
| 62 | NZ_CP021774.1 | GCA_002192475.1 | 17.14 Kb | 801,044 - 818,179 |
| 63 | NZ_CP173023.1 | GCA_044759025.1 | 17.00 Kb | 670,435 - 687,437 |
| 64 | NZ_OX638564.1 | GCA_951805275.2 | 17.00 Kb | 674,279 - 691,281 |
| 65 | NZ_CP171918.1 | GCA_053360635.1 | 29.26 Kb | 719,604 - 748,863 |
| 66 | NZ_CP155048.1 | GCA_023572735.3 | - | - |
| 67 | NZ_CP124657.1 | GCA_029961625.1 | 17.14 Kb | 742,413 - 759,548 |
| 68 | NZ_CP199841.1 | GCA_052697425.1 | 17.13 Kb | 680,926 - 698,056 |
| 69 | NZ_CP124655.1 | GCA_029961645.1 | 17.14 Kb | 6,176,967 - 6,194,102 |
| 70 | NZ_CP195322.1 | GCA_051257835.1 | 29.14 Kb | 6,204,661 - 6,233,797 |
| 71 | NZ_CP199192.1 | GCA_054127345.1 | 30.89 Kb | 745,782 - 776,667 |
| 72 | NZ_CP137498.1 | GCA_033223225.1 | 29.20 Kb | 5,088,593 - 5,117,791 |
| 73 | NZ_CP064401.1 | GCA_019857345.1 | 17.00 Kb | 741,342 - 758,345 |
| 74 | NZ_CP199200.1 | GCA_054129965.1 | 17.13 Kb | 727,655 - 744,785 |
| 75 | NZ_CP115482.1 | GCA_036237115.1 | 17.13 Kb | 1,720,414 - 1,737,542 |
| 76 | NZ_CP115273.1 | GCA_036233515.1 | 17.13 Kb | 2,998,654 - 3,015,782 |
| 77 | NZ_CP089236.1 | GCA_021184245.1 | 29.26 Kb | 723,168 - 752,427 |
| 78 | NZ_CP143357.1 | GCA_036326305.1 | 30.89 Kb | 6,260,770 - 6,291,657 |
| 79 | NZ_CP080289.1 | GCA_019434235.1 | 17.00 Kb | 737,525 - 754,528 |
| 80 | NZ_CP181666.1 | GCA_050186595.1 | 17.00 Kb | 692,796 - 709,798 |
| 81 | NZ_CP171953.1 | GCA_053360705.1 | 29.26 Kb | 704,343 - 733,602 |
| 82 | NZ_CP171901.1 | GCA_053372455.1 | 29.26 Kb | 718,106 - 747,365 |
| 83 | NZ_CP199764.1 | GCA_052695645.1 | 29.20 Kb | 677,942 - 707,141 |
| 84 | NZ_CP078004.1 | GCA_019857585.1 | 17.00 Kb | 737,531 - 754,534 |
| 85 | NZ_CP104584.1 | GCA_025398075.1 | 17.13 Kb | 726,458 - 743,588 |
| 86 | NZ_CP147625.1 | GCA_037287545.1 | 31.02 Kb | 763,980 - 794,997 |
| 87 | NZ_AP040349.1 | GCA_053343265.1 | 29.13 Kb | 682,836 - 711,965 |
| 88 | NZ_CP195317.1 | GCA_051257855.1 | 29.14 Kb | 1,889,990 - 1,919,126 |
| 89 | NZ_CP053747.1 | GCA_021378395.1 | 17.14 Kb | 768,386 - 785,520 |
| 90 | NZ_CP068678.1 | GCA_021513295.1 | 31.02 Kb | 2,191,666 - 2,222,683 |
| 91 | NZ_CP060242.1 | GCA_015697645.1 | 17.00 Kb | 1,141,203 - 1,158,206 |
| 92 | NZ_CP008865.2 | GCA_001515845.2 | 17.13 Kb | 683,815 - 700,945 |
| 93 | NZ_LR898867.1 | GCA_905071885.1 | 17.00 Kb | 672,516 - 689,520 |
| 94 | NZ_CP033771.1 | GCA_003812165.1 | 17.13 Kb | 5,506,418 - 5,523,548 |
| 95 | NZ_CP195320.1 | GCA_051257865.1 | 29.14 Kb | 5,334,841 - 5,363,977 |
| 96 | NZ_CP159841.1 | GCA_040562075.1 | 17.00 Kb | 1,774,135 - 1,791,138 |
| 97 | NZ_CP163150.1 | GCA_050239315.1 | 17.00 Kb | 702,067 - 719,070 |
| 98 | NZ_CM129832.1 | GCA_053096165.1 | 18.89 Kb | 741,881 - 760,769 |
| 99 | NZ_CP199791.1 | GCA_052695715.1 | 17.13 Kb | 683,688 - 700,818 |
| 100 | NZ_CP136539.1 | GCA_032811685.1 | 17.11 Kb | 677,171 - 694,279 |

**Supplementary Table 4:** 60 non-*P. aeruginosa*, *Pseudomonas* genomes (comprising *P. putida*, *P. protegens*, *P. chlororaphis* and *P. fluorescens*) containing tailocins for analysis via TattleTail

| **#** | **Genbank/Refseq** | **Region Length** | **Region Position** |
| --- | --- | --- | --- |
| 1 | NZ_CP016634.1 | - | - |
| 2 | NZ_CP136583.1 | - | - |
| 3 | NZ_CP054871.1 | - | - |
| 4 | NZ_CP054869.1 | - | - |
| 5 | NZ_CP054870.1 | - | - |
| 6 | NZ_CP054872.1 | - | - |
| 7 | NZ_CP054873.1 | - | - |
| 8 | NZ_AP024503.1 | - | - |
| 9 | NZ_CP051673.1 | - | - |
| 10 | NZ_CP043179.1 | - | - |
| 11 | AP045182.1 | - | - |
| 12 | NZ_CP013184.1 | - | - |
| 13 | NZ_CP147777.1 | - | - |
| 14 | NZ_CP195076.1 | - | - |
| 15 | NZ_CP031396.1 | - | - |
| 16 | NZ_CP171611.1 | - | - |
| 17 | NZ_CP022097.2 | - | - |
| 18 | NZ_CP195082.1 | - | - |
| 19 | NZ_CP063455.1 | - | - |
| 20 | NZ_CP081490.1 | - | - |
| 21 | NZ_CP054867.1 | - | - |
| 22 | NZ_CP054866.1 | - | - |
| 23 | NZ_CP019399.1 | - | - |
| 24 | NZ_JAQZQX010000001.1 | - | - |
| 25 | NZ_JAQZQQ010000001.1 | - | - |
| 26 | NZ_JAQZQV010000001.1 | - | - |
| 27 | NZ_JAQZQU010000001.1 | - | - |
| 28 | NZ_CP061079.1 | - | - |
| 29 | NZ_CP008696.1 | - | - |
| 30 | NZ_CP118156.1 | - | - |
| 31 | NZ_CP118147.1 | - | - |
| 32 | NZ_CP118140.1 | - | - |
| 33 | NZ_CP118137.1 | - | - |
| 34 | NZ_CP118155.1 | - | - |
| 35 | NZ_CP118154.1 | - | - |
| 36 | NZ_CP118142.1 | - | - |
| 37 | NZ_CP118143.1 | - | - |
| 38 | NZ_CP118145.1 | - | - |
| 39 | NZ_CP118141.1 | - | - |
| 40 | NZ_CP118144.1 | - | - |
| 41 | NZ_CM177065.1 | - | - |
| 42 | CP027561.1 | - | - |
| 43 | CP028826.1 | - | - |
| 44 | CP025542.1 | - | - |
| 45 | NZ_CP160329.1 | - | - |
| 46 | NZ_CP100660.1 | - | - |
| 47 | NZ_CP128260.1 | - | - |
| 48 | NZ_CP071797.1 | - | - |
| 49 | NZ_CP151816.1 | - | - |
| 50 | CP185740.1 | - | - |
| 51 | NZ_CP015638.1 | - | - |
| 52 | NZ_CP015637.1 | - | - |
| 53 | NZ_CDMF01000001.1 | - | - |
| 54 | NZ_CP063233.1 | - | - |
| 55 | NZ_CP104408.1 | - | - |
| 56 | CP022313.1 | - | - |
| 57 | NZ_UGUS01000002.1 | - | - |
| 58 | NZ_LS483372.1 | - | - |
| 59 | NZ_LT907842.1 | - | - |
| 60 | NZ_CP198989.1 | - | - |

**Supplementary Table 5:** 19 non-*P. aeruginosa* genomes for analysis via TattleTail

| **#** | **Genbank/Refseq** | **Assembly** | **Region Length** | **Region Position** |
| --- | --- | --- | --- | --- |
| 1 | CM000725.1 | GCA_000161095.1 | - | - |
| 2 | CM000758.1 | GCF_000161715.1 | - | - |
| 3 | AM920437.1 | GCA_000226395.1 | - | - |
| 4 | JACVVK020000003.1 | GCA_018292915.2 | - | - |
| 5 | JAVFKD010000014.1 | GCA_036429605.1 | - | - |
| 6 | JAQQWG010000004.1 | GCA_038362765.1 | - | - |
| 7 | BX950851.1 | GCF_000011605.1 | - | - |
| 8 | CM000748.1 | GCF_000161515.1 | - | - |
| 9 | CM000752.1 | GCF_000161595.1 | - | - |
| 10 | CM000753.1 | GCF_000161615.1 | - | - |
| 11 | CM000756.1 | GCF_000161675.1 | - | - |
| 12 | FO834906.1 | GCF_000968155.1 | - | - |
| 13 | CP015281.1 | GCF_001620335.1 | - | - |
| 14 | AP019314.1 | GCF_003945305.1 | - | - |
| 15 | CP098234.1 | GCF_029224125.1 | - | - |
| 16 | AP035801.1 | GCF_042847795.1 | - | - |
| 17 | FBYD01000004.1 | GCF_900041715.1 | - | - |
| 18 | LT599825.1 | GCF_900093485.1 | - | - |
| 19 | LR586051.1 | [GCA_901538445.1](https://www.ncbi.nlm.nih.gov/datasets/genome/GCA_901538445.1/) | - | - |

**Supplementary Table 6:** PHASTEST prediction of prophage regions in the genomes of the reference strains *P. aeruginosa* ATCC 27853, *P. aeruginosa* PAO1 and clinical *P. aeruginosa* isolate draft genomes.

| **Bacterial Strain** | **Region** | **Region Length** | **Completeness** | **Score** | **Region position** |
| --- | --- | --- | --- | --- | --- |
| *P. aeruginosa* ATCC 27853# | 1* | 18.4 Kb | Intact | 130 | 679,586 - 698,056 |
|  | 2 | 40.4 Kb | Intact | 114 | 796,824 - 837,266 |
|  | 3 | 42.9 Kb | Intact | 110 | 1,337,187 - 1,380,105 |
|  | 4 | 55.2 Kb | Questionable | 84 | 2,508,737 - 2,564,035 |
|  | 5 | 49.9 Kb | Intact | 150 | 5,102,870 - 5,152,806 |
|  | 6 | 15.1 Kb | Questionable | 85 | 5,787,383 - 5,802,527 |
| *P. aeruginosa* PAO1 | 1* | 35 Kb | Intact | 150 | 6,70,089 - 7,05,133 |
|  | 2 | 15.7 Kb | Intact | 107 | 7,80,995 - 7,96,776 |
| *P. aeruginosa* 003 | 1 | 57.1 Kb | Intact | 150 | 3,283,954 - 3,341,094 |
|  | 2 | 38.3 Kb | Intact | 150 | 6,203,998 - 6,242,311 |
|  | 3* | 18.4 Kb | Intact | 140 | 6,283,681 - 6,302,155 |
| *P. aeruginosa* 005 | 1 | 21.4 Kb | Questionable | 90 | 137,662 - 159120 |
|  | 2* | 18.9 Kb | Questionable | 80 | 3,827,311 - 3,846,308 |
| *P. aeruginosa* 012 | 1* | 35 Kb | Intact | 150 | 401,663 - 436,707 |
|  | 2 | 45.1 Kb | Intact | 110 | 2,442,251 - 2,483,375 |

* Manual inspection during this study revealed that these regions fulfilled conserved criteria of pyocin-encoding gene clusters in *P. aeruginosa* and not that of prophage-encoding regions.

\# Manual inspection was also performed as part of a previous study (Pais et al. 2026, https://doi.org/10.1099/mic.0.001692) and was redone in this study since *P. aeruginosa* ATCC 27853 serves as a reference strain.

**Supplementary Table 7:** Phigaro prediction of prophage regions in the genome of the reference strains *P. aeruginosa* ATCC 27853, *P. aeruginosa* PAO1 and clinical *P. aeruginosa* isolate draft genomes.

| **Bacterial Strain** | **Region** | **Taxonomy** | **Region position** |
| --- | --- | --- | --- |
| *P. aeruginosa* ATCC 27853# | 1* | Myoviridae | 677,789 – 698,888 |
|  | 2 | Siphoviridae | 796,823 – 837,265 \| |
|  | 3 | Siphoviridae | 1,350,632 – 1,375,437 |
|  | 4 | Siphoviridae | 2,508,910 – 2,560,414 |
|  | 5 | Myoviridae | 5,098,936 – 5,130,515 |
|  | 6 | Siphoviridae | 5,514,466 – 5,525,918 |
|  | 7 | Inoviridae | 5,784,505 – 5,794,381 |
| *P. aeruginosa* PAO1 | 1* | Myoviridae | 670,979 – 704,081 |
| *P. aeruginosa* 003 | 1 | Siphoviridae | 3,299,257 - 3343154 |
|  | 2 | Myoviridae | 6,205,589 – 6,246,876 |
|  | 3* | Myoviridae | 6,282,015 – 6,302,987 |
| *P. aeruginosa* 005 | 1 | Myoviridae | 141,151 – 159,502 |
|  | 2 | Unknown | 1,017,789 – 1,031,454 |
|  | 3* | Siphoviridae | 3,827,310 – 3,847,972 |
| *P. aeruginosa* 012 | 1* | Myoviridae | 402,713 – 435,815 |
|  | 2 | Siphoviridae | 2,444,012 – 2,483,374 |

* Manual inspection during this study revealed that these regions fulfilled conserved criteria of pyocin-encoding gene clusters in *P. aeruginosa* and not that of prophage-encoding regions.

\# Manual inspection was also performed as part of a previous study (Pais et al. 2026, https://doi.org/10.1099/mic.0.001692) and was redone in this study since *P. aeruginosa* ATCC 27853 serves as a reference strain.

**Supplementary Table 8:** VirSorter prediction of prophage regions in the genome of the reference strains *P. aeruginosa* ATCC 27853, *P. aeruginosa* PAO1 and clinical *P. aeruginosa* isolate draft genomes.

| **Bacterial Strain** | **Region** | **Phage type** | **Score** | **Region position** |
| --- | --- | --- | --- | --- |
| *P. aeruginosa* ATCC 27853# | 1 | dsDNA phage | 0.9 | 2,507,824 – 2,670,334 |
|  | 2 | dsDNA phage | 0.9 | 1,333,871 – 1,445,915 |
|  | 3 | dsDNA phage | 0.927 | 5,092,954 – 5,196,790 |
|  | 4 | dsDNA phage | 0.913 | 795,599 – 890,791 |
|  | 5* | dsDNA phage | 0.9 | 679,586 – 713,091 |
| *P. aeruginosa* PAO1 | 1* | dsDNA phage | 0.953 | 672,777 – 732,094 |
| *P. aeruginosa* 003 | 1 | dsDNA phage | 0.927 | 3,285,067 – 3,410,885 |
|  | 2 | dsDNA phage | 0.913 | 6,200,441 – 6,270,136 |
|  | 3* | dsDNA phage | 0.967 | 6,282,016 – 6,306,642 |
| *P. aeruginosa* 005 | 1 | dsDNA phage | 0.9 | 120,606 – 178,748 |
|  | 2 | dsDNA phage | 0.927 | 1,017,790 – 1,035,830 |
| *P. aeruginosa* 012 | 1 | dsDNA phage | 0.92 | 2,442,251 – 2,556,346 |
|  | 2* | dsDNA phage | 0.873 | 395,616 – 457,866 |

* Manual inspection during this study revealed that these regions fulfilled conserved criteria of pyocin-encoding gene clusters in *P. aeruginosa* and not that of prophage-encoding regions.

\# Manual inspection was also performed as part of a previous study (Pais et al. 2026, https://doi.org/10.1099/mic.0.001692s) and was redone in this study since *P. aeruginosa* ATCC 27853 serves as a reference strain.

**Supplementary Figure 1:** Serial dilution spot tests of each representative predicted pyocin against *P. aeruginosa* 008. Spot tests in the upper row show activity of mitomycin C induced, TFF-processed pyocin samples. Spot tests in the bottom row show activity of uninduced, TFF-processed supernatants from each strain harbouring a representative pyocin.

**Supplementary Figure 2:** Serial dilution spot tests for necessary controls against *P. aeruginosa* 008. **(A)** Effect of Proteinase K on the efficacy of the pyocins to rule out S-type pyocin-attributed bactericidal effects. **(B)** Effect of endogenous pyocins on their respective producer strain to evaluate self-susceptibility. **(C)** Lack of replicative ability of the active agent/agents in TFF-processed pyocin samples.
