# Supplementary material for: TattleTail: A Rule-Based Tool for Identifying Canonical Pyocin-Encoding Gene Clusters in Pseudomonas aeruginosa
Source: Comput Struct Biotechnol J. 2026 Sep 30;35(1):0230. doi: 10.34133/csbj.0230 (PMC13624779; doi:10.34133/csbj.0230)
Supplement: Supplementary file 1 [file csbj.0230.f1.zip › Supplementary Folder 3.zip!/Supplementary Folder 3/Detailed Phigaro output/Phigaro P. aeruginosa 003/cgview.phigaro.html]

Phigaro report: input/cgview.fa


| # | Coordinates | Taxonomy | pVOGs |
| --- | --- | --- | --- |
| contig\_Unknown\_start\_1\_stop\_664419 | | | |
| --- | --- | --- | --- |
| 1 | 3299257 - 3343154 | Siphoviridae |  || Databases to Blast Against Nucleotide collection (nr/nt) \|; RefSeq Genome Database (refseq\_genomes) \| | | | |
| VOG0980, VOG4365, VOG4780, VOG1419, VOG1474, VOG1418, VOG2172, VOG2171, VOG2170, VOG9868, VOG2168, VOG2167, VOG5492, VOG2166, VOG2165, VOG0438, VOG3178, VOG8529, VOG4807, VOG0572, VOG4652, VOG1356, VOG4544, VOG4005, VOG4004, VOG4003, VOG4002, VOG8528, VOG4795, VOG10848, VOG4001, VOG3556, VOG3995, VOG3999, VOG10847, VOG3998, VOG1564, VOG3995, VOG3993, VOG4892, VOG8527, VOG8526, VOG8525, VOG3992, VOG4791, VOG6855, VOG2153, VOG2154 | | | |

| 2 | 6205589 - 6246876 | Myoviridae |  || Databases to Blast Against Nucleotide collection (nr/nt) \|; RefSeq Genome Database (refseq\_genomes) \| | | | |
| VOG7110, VOG0376, VOG2202, VOG10925, VOG2204, VOG2205, VOG11073, VOG7243, VOG2206, VOG4691, VOG2207, VOG2216, VOG0299, VOG4565, VOG8376, VOG0585, VOG2210, VOG2211, VOG2212, VOG2213, VOG0568, VOG0567, VOG0764, VOG0566, VOG3897, VOG4556, VOG0267, VOG8435, VOG3379, VOG6627, VOG0275, VOG0979, VOG0052, VOG4551 | | | |

| 3 | 6282015 - 6302987 | Myoviridae |  || Databases to Blast Against Nucleotide collection (nr/nt) \|; RefSeq Genome Database (refseq\_genomes) \| | | | |
| VOG8347, VOG2215, VOG0585, VOG0198, VOG0977, VOG0573, VOG4550, VOG4691, VOG0574, VOG6373, VOG2073, VOG0356, VOG0577, VOG8340, VOG4130, VOG4736, VOG0569, VOG0582, VOG3721, VOG6174, VOG6173 | | | |

For all prophage sequences:
