# Supplementary material for: TattleTail: A Rule-Based Tool for Identifying Canonical Pyocin-Encoding Gene Clusters in Pseudomonas aeruginosa
Source: Comput Struct Biotechnol J. 2026 Sep 30;35(1):0230. doi: 10.34133/csbj.0230 (PMC13624779; doi:10.34133/csbj.0230)
Supplement: Supplementary file 1 [file csbj.0230.f1.zip › Supplementary Folder 3.zip!/Supplementary Folder 3/Detailed Phigaro output/Phigaro P. aeruginosa 005/cgview.phigaro.html]

Phigaro report: input/cgview.fa


| # | Coordinates | Taxonomy | pVOGs |
| --- | --- | --- | --- |
| contig\_Unknown\_start\_1\_stop\_613768 | | | |
| --- | --- | --- | --- |
| 1 | 141151 - 159502 | Myoviridae |  || Databases to Blast Against Nucleotide collection (nr/nt) \|; RefSeq Genome Database (refseq\_genomes) \| | | | |
| VOG2167, VOG0976, VOG3434, VOG4573, VOG0833, VOG1933, VOG0051, VOG0831, VOG7152, VOG4645, VOG0615, VOG0376, VOG7106, VOG4693, VOG0355, VOG5994 | | | |

| 2 | 1017789 - 1031454 | Unknown |  || Databases to Blast Against Nucleotide collection (nr/nt) \|; RefSeq Genome Database (refseq\_genomes) \| | | | |
| VOG7465, VOG8348, VOG4658, VOG8349, VOG7884, VOG0719, VOG1309, VOG4600, VOG6865, VOG6864, VOG6413, VOG9482, VOG1960 | | | |

| 3 | 3827310 - 3847972 | Siphoviridae |  || Databases to Blast Against Nucleotide collection (nr/nt) \|; RefSeq Genome Database (refseq\_genomes) \| | | | |
| VOG0980, VOG4780, VOG5820, VOG4598, VOG4701, VOG0850, VOG0848, VOG0847, VOG4735, VOG3428, VOG3427, VOG6173, VOG6174, VOG3721, VOG0198, VOG0585, VOG2215, VOG8347 | | | |

For all prophage sequences:
