# Supplementary material for: TattleTail: A Rule-Based Tool for Identifying Canonical Pyocin-Encoding Gene Clusters in Pseudomonas aeruginosa
Source: Comput Struct Biotechnol J. 2026 Sep 30;35(1):0230. doi: 10.34133/csbj.0230 (PMC13624779; doi:10.34133/csbj.0230)
Supplement: Supplementary file 1 [file csbj.0230.f1.zip › Supplementary Folder 3.zip!/Supplementary Folder 3/Detailed Phigaro output/Phigaro P. aeruginosa 012/cgview.phigaro.html]

Phigaro report: input/cgview.fa


| # | Coordinates | Taxonomy | pVOGs |
| --- | --- | --- | --- |
| contig\_Unknown\_start\_1\_stop\_668136 | | | |
| --- | --- | --- | --- |
| 1 | 402713 - 435815 | Myoviridae |  || Databases to Blast Against Nucleotide collection (nr/nt) \|; RefSeq Genome Database (refseq\_genomes) \| | | | |
| VOG0980, VOG4365, VOG9340, VOG0980, VOG4365, VOG9340, VOG4598, VOG4701, VOG0850, VOG0848, VOG0847, VOG4735, VOG3428, VOG3427, VOG6173, VOG6174, VOG3721, VOG0582, VOG0569, VOG4736, VOG4130, VOG8340, VOG0577, VOG0356, VOG6373, VOG0574, VOG4691, VOG4550, VOG0573, VOG0977, VOG0198, VOG0585, VOG2215, VOG8347 | | | |

| 2 | 2444012 - 2483374 | Siphoviridae |  || Databases to Blast Against Nucleotide collection (nr/nt) \|; RefSeq Genome Database (refseq\_genomes) \| | | | |
| VOG2154, VOG7620, VOG2153, VOG6855, VOG6086, VOG4791, VOG3992, VOG8526, VOG8527, VOG4892, VOG3993, VOG3995, VOG10952, VOG3998, VOG10847, VOG3999, VOG3556, VOG4001, VOG10848, VOG4795, VOG4002, VOG6153, VOG4005, VOG4544, VOG1356, VOG10995, VOG0572, VOG2163, VOG6012, VOG2790, VOG2165, VOG2166, VOG5492, VOG2167, VOG2168, VOG11071, VOG2170, VOG2171, VOG2172, VOG1418, VOG1474, VOG1419, VOG4780, VOG4365, VOG0980 | | | |

For all prophage sequences:
