# Supplementary material for: TattleTail: A Rule-Based Tool for Identifying Canonical Pyocin-Encoding Gene Clusters in Pseudomonas aeruginosa
Source: Comput Struct Biotechnol J. 2026 Sep 30;35(1):0230. doi: 10.34133/csbj.0230 (PMC13624779; doi:10.34133/csbj.0230)
Supplement: Supplementary file 1 [file csbj.0230.f1.zip › Supplementary Folder 3.zip!/Supplementary Folder 3/Detailed Phigaro output/Phigaro P. aeruginosa ATCC 27853/cgview.phigaro.html]

Phigaro report: input/cgview.fa


| # | Coordinates | Taxonomy | pVOGs |
| --- | --- | --- | --- |
| NZ\_CP101912\_1 | | | |
| --- | --- | --- | --- |
| 1 | 677789 - 698888 | Myoviridae |  || Databases to Blast Against Nucleotide collection (nr/nt) \|; RefSeq Genome Database (refseq\_genomes) \| | | | |
| VOG8347, VOG2215, VOG0585, VOG0198, VOG0977, VOG0573, VOG4550, VOG4691, VOG0574, VOG10969, VOG2073, VOG0356, VOG0577, VOG8340, VOG4130, VOG4736, VOG0569, VOG0582, VOG3721, VOG6174, VOG6173 | | | |

| 2 | 796823 - 837265 | Siphoviridae |  || Databases to Blast Against Nucleotide collection (nr/nt) \|; RefSeq Genome Database (refseq\_genomes) \| | | | |
| VOG2154, VOG7620, VOG2153, VOG6855, VOG6086, VOG4791, VOG3992, VOG8525, VOG5531, VOG5472, VOG8527, VOG4892, VOG3993, VOG3995, VOG10952, VOG3998, VOG10847, VOG3999, VOG3556, VOG4001, VOG10848, VOG4795, VOG4002, VOG6153, VOG4005, VOG4544, VOG1356, VOG10995, VOG0572, VOG2163, VOG6012, VOG2790, VOG2165, VOG2166, VOG5492, VOG2167, VOG2168, VOG11071, VOG2170, VOG2171, VOG2172, VOG1418, VOG1474, VOG1419, VOG4780, VOG0980, VOG4694 | | | |

| 3 | 1350632 - 1375437 | Siphoviridae |  || Databases to Blast Against Nucleotide collection (nr/nt) \|; RefSeq Genome Database (refseq\_genomes) \| | | | |
| VOG10933, VOG5084, VOG4851, VOG0355, VOG0296, VOG2096, VOG0615, VOG0300, VOG7152, VOG0975, VOG0051, VOG4361, VOG0833, VOG4672, VOG4364, VOG5492, VOG2167, VOG7243 | | | |

| 4 | 2508910 - 2560414 | Siphoviridae |  || Databases to Blast Against Nucleotide collection (nr/nt) \|; RefSeq Genome Database (refseq\_genomes) \| | | | |
| VOG0275, VOG9547, VOG8348, VOG3449, VOG0244, VOG5531, VOG8349, VOG4548, VOG0083, VOG7884, VOG0719, VOG1309, VOG4600, VOG1636, VOG7473, VOG6865, VOG6864, VOG6413, VOG9482, VOG2215, VOG4566, VOG0025, VOG4693, VOG7106, VOG2388, VOG2125, VOG0796, VOG4544, VOG7470, VOG2464, VOG5027, VOG2466, VOG5296, VOG2467, VOG6400, VOG2468, VOG2469, VOG0697, VOG2471, VOG5601, VOG2473, VOG8171, VOG5277, VOG1539, VOG3179, VOG4743, VOG7568, VOG3447, VOG4547, VOG6174, VOG6173, VOG3448 | | | |

| 5 | 5098936 - 5130515 | Myoviridae |  || Databases to Blast Against Nucleotide collection (nr/nt) \|; RefSeq Genome Database (refseq\_genomes) \| | | | |
| VOG10038, VOG0189, VOG4556, VOG3897, VOG0566, VOG0764, VOG0567, VOG0568, VOG0569, VOG2388, VOG2125, VOG4616, VOG4645, VOG0571, VOG0572, VOG0573, VOG4550, VOG4691, VOG0574, VOG6373, VOG2073, VOG0356, VOG0577, VOG4630, VOG4736, VOG0582, VOG3556, VOG2186, VOG0583 | | | |

| 6 | 5514466 - 5525918 | Siphoviridae |  || Databases to Blast Against Nucleotide collection (nr/nt) \|; RefSeq Genome Database (refseq\_genomes) \| | | | |
| VOG4791, VOG5562, VOG5998, VOG0025, VOG2039, VOG2485, VOG9481, VOG4694, VOG0267, VOG1931 | | | |

| 7 | 5784505 - 5794381 | Inoviridae |  || Databases to Blast Against Nucleotide collection (nr/nt) \|; RefSeq Genome Database (refseq\_genomes) \| | | | |
| VOG0083, VOG0275, VOG0275, VOG4757, VOG4757 | | | |

For all prophage sequences:
