# Supplementary material for: TattleTail: A Rule-Based Tool for Identifying Canonical Pyocin-Encoding Gene Clusters in Pseudomonas aeruginosa
Source: Comput Struct Biotechnol J. 2026 Sep 30;35(1):0230. doi: 10.34133/csbj.0230 (PMC13624779; doi:10.34133/csbj.0230)
Supplement: Supplementary file 1 [file csbj.0230.f1.zip › Supplementary Folder 3.zip!/Supplementary Folder 3/Detailed Phigaro output/Phigaro P. aeruginosa PAO1/cgview.phigaro.html]

Phigaro report: input/cgview.fa


| # | Coordinates | Taxonomy | pVOGs |
| --- | --- | --- | --- |
| NC\_002516\_2 | | | |
| --- | --- | --- | --- |
| 1 | 670979 - 704081 | Myoviridae |  || Databases to Blast Against Nucleotide collection (nr/nt) \|; RefSeq Genome Database (refseq\_genomes) \| | | | |
| VOG8347, VOG2215, VOG0585, VOG0198, VOG0977, VOG0573, VOG4550, VOG4691, VOG0574, VOG6373, VOG0356, VOG0577, VOG8340, VOG4130, VOG4736, VOG0569, VOG0582, VOG3721, VOG6174, VOG6173, VOG3427, VOG3428, VOG4735, VOG0847, VOG0848, VOG0850, VOG4701, VOG4598, VOG9340, VOG4365, VOG0980, VOG9340, VOG4365, VOG0980 | | | |

For all prophage sequences:
